# A New Trichlorinated Xanthone and Compounds Isolated from *Cladonia skottsbergii* with Antimicrobial Properties

**DOI:** 10.3390/ph19010174

**Published:** 2026-01-19

**Authors:** Marvin J. Rositzki, Achara Raksat, Charles J. Simmons, Clifford Smith, Reverend Danette V. Choi, Supakit Wongwiwatthananukit, Leng Chee Chang

**Affiliations:** 1 Department of Pharmaceutical Sciences, The Daniel K. Inouye College of Pharmacy, University of Hawai’i at Hilo, Hilo, HI 96720, USA; rositzki@hawaii.edu (M.J.R.); achara@hawaii.edu (A.R.); 2 Department of Pharmacy Practice, The Daniel K. Inouye College of Pharmacy, University of Hawai’i at Hilo, Hilo, HI 96720, USA; supakit@hawaii.edu; 3 X-Ray Diffraction Laboratory, Department of Chemistry, University of Hawai’i at Hilo, Hilo, HI 96720, USA; simmonsc@hawaii.edu; 4 Department of Botany, University of Hawai’i at Manoa, Honolulu, HI 96822, USA; cliff@hawaii.edu; 5 Bang San Ho Temple Yun Hwa Denomination of World Social Buddhism dba Lotus Buddhist Monastery, Mountain View, HI 96771, USA; manjin@yunhwasangha.org; 6 College of Pharmacy, Rangsit University, Pathum Thani 12000, Thailand

**Keywords:** *C. skottsbergii*, lichen, chlorinated xanthone, MRSA, MSSA, Hawaii, antibacterial activity

## Abstract

**Background/Objectives**: The global rise in multidrug-resistant (MDR) bacteria, particularly methicillin-resistant and methicillin-susceptible *Staphylococcus aureus* (MRSA and MSSA), continues to pose a major public health challenge, including in Hawaii. This underscores the need to discover new antimicrobial agents from natural sources. Guided by teachings from a Buddhist master regarding the medicinal value of lichens, we investigated the endemic Hawaiian lichen *Cladonia skottsbergii*. **Methods**: Specimens of *C. skottsbergii* were collected from the Lotus Buddhist Monastery in Mountain View, Hawaii. A methanolic extract was prepared and purified using chromatographic techniques, and compound structures were elucidated through spectroscopic analyses and single-crystal X-ray diffraction. The antibacterial activity of the compounds was assessed against Gram-positive strains (MRSA, MSSA) and Gram-negative bacteria (*Escherichia coli*, *Klebsiella pneumoniae*, *Pseudomonas aeruginosa*). Cytotoxicity was assessed using A549 (non-small cell lung cancer) and Vero E6 (non-tumorigenic) cell lines. **Results**: Three compounds were isolated: clarosione (**1**), a newly identified trichlorinated xanthone, and two known metabolites, (*S*)-usnic acid (**2**) and perlatolic acid (**3**). Compounds **2** and **3** demonstrated strong inhibitory effects against MRSA and MSSA. Their minimum inhibitory concentrations (MICs) ranged from 2 to 4 µg/mL, compared with vancomycin (0.5–1 µg/mL). Cytotoxicity testing showed higher sensitivity in A549 cells than in Vero E6 cells, resulting in favorable selectivity indices for the active compounds. **Conclusions**: In the current study, a new compound, clarosione (**1**) was discovered. This enhances our understanding of the constituents of *C. skottsbergii* and its potential antibacterial properties. Lichen-derived compounds may serve as lead candidates for further development, and further study is warranted.

## 1. Introduction

The global crisis of multidrug-resistant (MDR) bacteria is forecasted to result in 10 million annual fatalities by 2050 [1,2]. The threat has been intensified by the COVID-19 pandemic, during which hospitalized patients faced heightened vulnerability to secondary infections from both methicillin-resistant and methicillin-susceptible *Staphylococcus aureus* (MRSA and MSSA) [3]. These infections contribute significantly to prolonged hospitalizations and higher mortality rates, with incidence in Hawaii exceeding the U.S. average [4]. Both MRSA and MSSA are major causes of pneumonia, a leading cause of death worldwide. Community-acquired and healthcare-associated MRSA pneumonia are particularly challenging to treat due to the presence of antibiotic-resistance genes [5]. Gram-positive bacteria, such as MRSA, exhibit resistance to *β*-lactam antibiotics by disrupting cell wall synthesis mechanisms [6]. Therefore, addressing antibiotic resistance is essential to reducing infection-related mortality.

The growing threat of MRSA underscores the urgent need for new antimicrobial drugs. Natural products are a viable source of therapeutic compound discovery [7]. Lichens, symbiotic organisms composed of algae and fungi, are particularly rich in secondary metabolites with diverse bioactivities, including antifungal, anticancer, and antiviral properties [8]. Many lichen-derived compounds, such as usnic acid, exhibit potent antibacterial and anti-inflammatory effects, useful for treating respiratory and skin [9,10,11]. However, lichens are highly sensitive to air pollution and rarely grow in industrial or urban areas, especially where sulfur dioxide is present [12].

**Figure 1:**
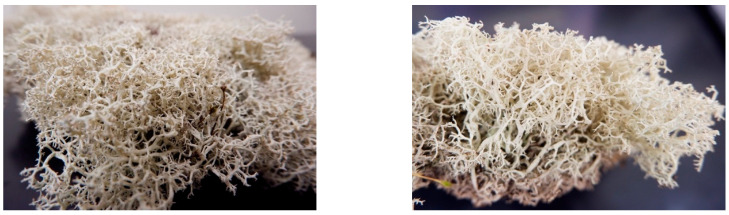
*Cladonia skottsbergii*.

Hawaii’s unique environment supports diverse lichen species due to low pollution, high humidity, and extended rainy season (approximately 7.3 months), with average annual rainfall of 230 cm and temperatures ranging from 15 °C to 26.7 °C [13]. Among these, *Cladonia skottsbergii* (Figure 1) is found primarily on the Big Island, particularly in the Mountain View (Puna district), where it was collected at the Lotus Buddhist Monastery. It also grows in geothermal areas such as Puhimau and near Kilauea Volcano [14,15]. Despite favorable ecological conditions, few natural products have been reported from *Cladonia* species [16,17,18].

This study aims to isolate and characterize natural products derived from *C. skottsbergii* extracts, evaluate their antimicrobial activity against Gram-positive and Gram-negative bacteria, and determine their cytotoxic properties.

## 2. Results and Discussion

### 2.1. Structure Elucidation

**Figure 2:**
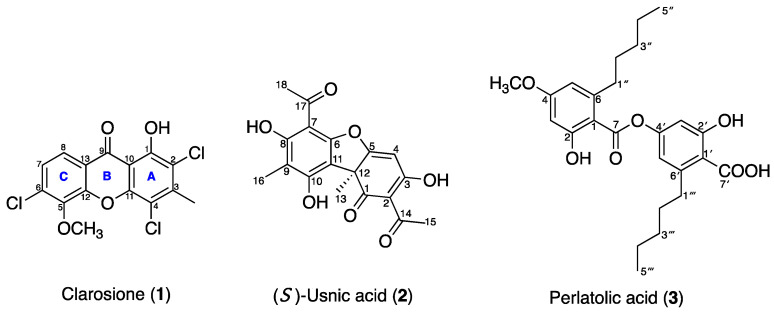
Structures of compounds (**1**–**3**) from *C. skottsbergii* methanolic extract.

The methanolic extract of *C. skottsbergii* was purified using column chromatography, which successfully yielded three compounds: a new chlorinated xanthone named clarosione (**1**), along with two known compounds, (*S*)-usnic acid (**2**) [19] and perlatolic acid (**3**) (Figure 2) [20]. The identification of the known compounds involved comparing their physical and spectroscopic data with published values. The structure of the new compound was elucidated based on NMR spectroscopy and single-crystal X-ray diffraction data.

**Figure 3:**
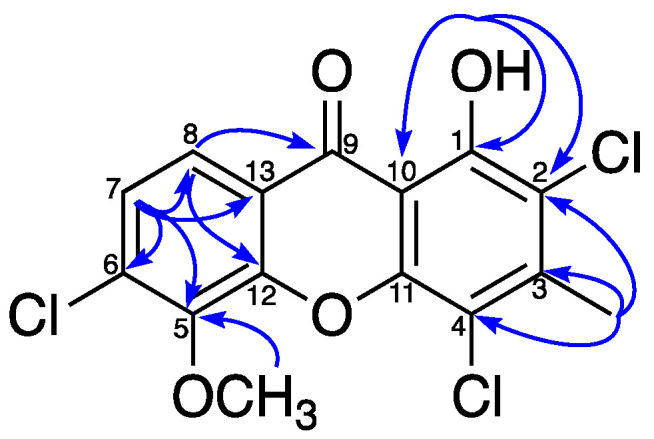
Key HMBC correlations for clarosione (**1**).

**Figure 4:**
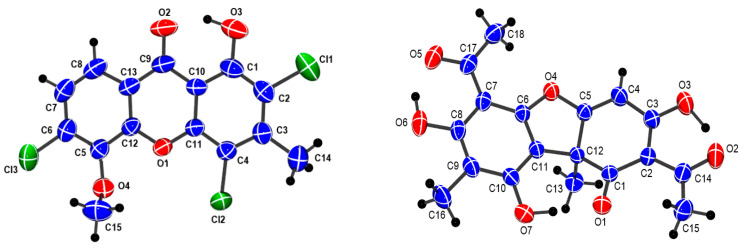
Drawing of clarosione (**1**) (C_15_H_9_Cl_3_O_4_) (**left**) and one of the two (*S*)-usnic acid (**2**) (C_18_H_16_O_7_) molecules in the asymmetric unit (**right**) measured at 298 K. Atoms in both structures are drawn with displacement ellipsoids at the 50% probability level.

Clarosione (**1**) was obtained as a yellow needle and gave an ion peak [M + H]^+^ at *m*/*z* 358.9635 in its HRESIMS, corresponding to a molecular formula of C_15_H_9_Cl_3_O_4_ (calcd. for C_15_H_10_Cl_3_O_4_, *m*/*z* 358.9639) (Figure S6). The infrared (IR) spectrum revealed stretching bands for carbonyl groups at 1690 cm^−1^ and C–Cl groups at 817 cm^−1^ (Figure S7), while the ultraviolet (UV) spectrum exhibited absorption bands at *λ*_max_ 210 nm and 280 nm (Figure S8), which are characteristic of a xanthone chromophore [21,22]. The ^1^H and ^13^C NMR data (Table 1, Figures S1 and S2) displayed resonances for a H-bonded hydroxy proton [*δ*_H_ 13.08 (1H, s, OH–1)], two ortho-coupled aromatic protons [*δ*_H_/*δ*_C_ 7.94 (1H, d, *J* = 8.7 Hz, H–8)/120.8 and 7.44 (1H, d, *J* = 8.7 Hz, H–7)/126.1], one methyl group [*δ*_H_/*δ*_C_ 2.67 (1H, s, 3–CH_3_)/19.0], and one methoxy group [*δ*_H_/*δ*_C_ 4.20 (1H, s, 5–OCH_3_/61.7]. The H–H-bonded hydroxy proton (*δ*_H_ 13.08) was attached at C–1 (*δ*_C_ 155.3), and the observed correlation from methyl protons (*δ*_H_ 2.67) with C–2 (*δ*_C_ 116.4), C–3 (*δ*_C_ 144.7), and C–4 (*δ*_C_ 112.2) confirmed the position of 3–CH_3_ (Figure 3). The methoxyl group was positioned at C–5 based on HMBC correlations between the methoxyl protons (*δ*_H_ 4.20) and C–5 (*δ*_C_ 145.2). The HMBC correlations of H–7 (*δ*_H_ 7.44) with C–5 (*δ*_C_ 145.2), C–6 (*δ*_C_ 135.5), C–8 (*δ*_C_ 120.8), C–13 (*δ*_C_ 119.9), and H–8 (*δ*_H_ 7.94) with C–9 (*δ*_C_ 180.7) and C–12 (*δ*_C_ 150.1) confirmed the location of a set of ortho-coupled aromatic protons at C–7 and C–8 (Figures S3–S5). The positions of three chlorine atoms were confirmed by single-crystal X-ray analysis (Figure 4), and the structure is **1**. The first chlorine-containing xanthone, 2-chloronorlichexanthone, was found in 1966 in the lichen *Lecanora rupicola* by Huneck [23]. Clarosione (**1**) is a rare xanthone. It contains three chlorine atoms—two atoms on the A ring and one on the C ring (Figure 2).

Only one report (2014) described an uncommon chlorinated xanthone, 1,5-dihydroxy-2,4,6-trichloro-7-methylxanthone, from the lichen *Cladonia incrassate;* it demonstrated weaker antimicrobial activity against *S. aureus* than (-)-usnic acid [24].

**Figure 5:**
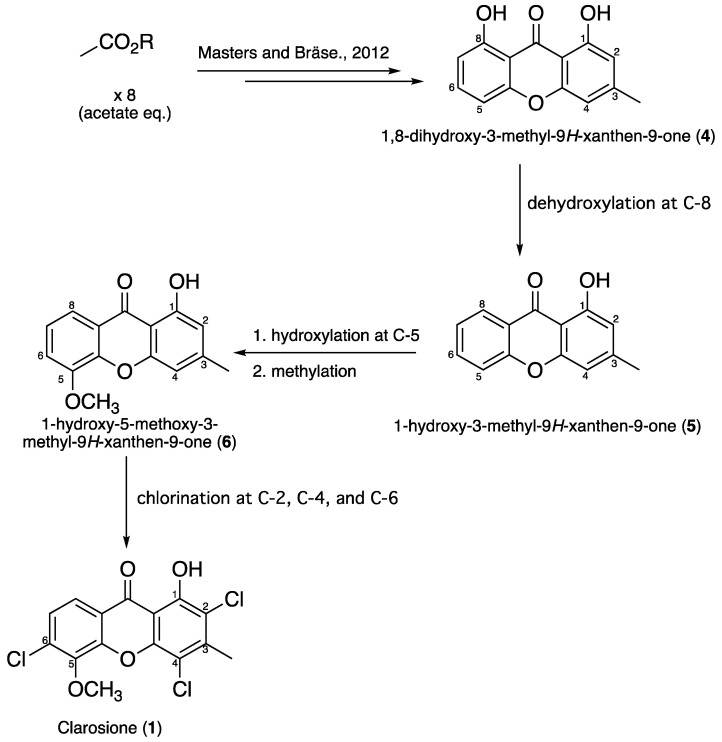
Plausible biosynthetic pathways for clarosione (**1**) [25].

A proposed biosynthetic pathway for clarosione (**1**) is illustrated in Figure 5. Accordingly, the process begins with the dehydroxylation of compound **4** (1,8-dihydroxy-3-methyl-9H-xanthen-9-one) [25] at carbon 8, resulting in the formation of 1-hydroxy-3-methyl-9H-xanthen-9-one (**5**). Next, selective hydroxylation occurs at carbon 5 of compound **5**, followed by methylation, producing 1-hydroxy-5-methoxy-3-methyl-9H-xanthen-9-one (**6**). Finally, chlorination at carbons 2, 4, and 6 of compound **6** leads to the formation of clarosione (**1**).

(*S*)-Usnic acid (**2**) is a dibenzofuran derivative isolated as greenish-yellow needles. Its molecular formula, C_18_H_16_O_7_, was assigned based on NMR spectroscopic data [19]. The ^1^H and ^13^C NMR spectra displayed one aromatic proton at *δ*_H_/*δ*_C_ 5.98 (1H, s, 4–H)/98.5, two methyls at 2.11 (3H, s, 16–CH_3_)/7.7 and 1.76 (3H, s, 12–CH_3_)/32.3, and resonances of two acetoxy groups at *δ*_H_/*δ*_C_ 2.68 (3H, s, 2–OCCH_3_)/28.0 and *δ*_C_ 201.8, 2.67 (3H, s, 7–OCCH_3_)/31.4 with *δ*_C_ 200.4 (Figures S9 and S10). In analyzing the HMBC correlations, the assignment of methyl substituents at C–9 and C–12, was supported: specifically, *δ*_H_ 2.11 showed a correlation with C–9 (*δ*_C_ 109.6), while *δ*_H_ 1.76 correlated with C–12 (*δ*_C_ 59.2). Furthermore, the locations of the two acetoxy groups were confirmed through HMBC, with *δ*_H_ 2.68 correlated with C–2 (*δ*_C_ 105.5) and *δ*_H_ 2.67 correlated with C–7 (*δ*_C_ 101.7) (Figures S11–S14). The absolute configuration of the chiral center at C–12 of **2** was confirmed as the *S*-configuration by single-crystal X-ray diffraction data (Figure 4), and its specific rotation was −472 (c 0.1, CHCl_3_) (*R*–configuration at +478 [26]). Usnic acid (**2**) is a common lichen metabolite with biological activities [10]. Both the (+)- and (-)-enantiomers of usnic acid have been found in lichens, with the (+)-enantiomer being more prevalent in *Cladonia* (*Cladina*), *Parmelia*, *Ramalina*, and *Usnea* species, while the (-)-enantiomer predominates in most species of *Alectoria*, *Cladonia*, *Rhizoplaca*, and several other genera [27]. (The two enantiomers differ in the –CH_3_ group at the chiral C–12 position projected in their *R* or *S* configuration [28]).

Perlatolic acid (**3**), a depside derivative with the molecular formula C_25_H_32_O_7_ [20], was isolated as a white solid, and its structure was determined by ^1^H and ^13^C NMR spectro-scopic data. NMR spectra displayed resonances for a H-bonded hydroxy proton at *δ*_H_ 11.36 (1H, s, OH–1), two sets of aromatic meta coupling protons at *δ*_H_/*δ*_C_ 6.74 (1H, d, *J* = 2.5 Hz, 3′–H)/109.1, 6.62 (1H, d, *J* = 2.5 Hz, 5′–H)/116.2, 6.38 (1H, m, 3–H)/99.1, and 6.37 (1H, m, 5–H)/111.6, two sets of pentyl groups at *δ*_H_/*δ*_C_ 2.99 (2H, m, 1‴–H_2_)/36.7, 2.96 (2H, m, 1″–H_2_)/37.4, 1.65 (2H, m, 3″–H_2_)/32.1, 1.64 (2H, m, 3‴–H_2_)/31.6, 1.37 (2H, m, 4″–H_2_)/22.7, 1.36 (2H, m, 2″–H_2_)/32.2, 1.36 (2H, m, 4‴–H_2_)/22.6, 1.33 (2H, m, 2‴–H_2_)/32.2, 0.91 (3H, t, *J* = 7.2 Hz, 5″–CH_3_)/14.2, and 0.87 (3H, t, *J* = 7.2 Hz, 5‴–CH_3_)/14.2, one methoxy group at *δ*_H_/*δ*_C_ 3.84 (3H, s, 4–OCH_3_)/55.6, one carbonyl at *δ*_C_ 169.6 (C–7), and one carboxylic unit at *δ*_C_ 174.1 (C–7’) (Figures S15 and S16). Two pentyl substituents were assigned to C–6 and C–1′ based on HMBC correlations. The cross-peaks observed between H_2_–1″ (*δ*_H_ 2.96) and C–1 (*δ*_C_ 103.5), C–6 (*δ*_C_ 148.5), and C–5 (*δ*_C_ 111.6) established the attachment of the first pentyl group at C–6. Likewise, correlations of H_2_–1‴ with C–1′ (*δ*_C_ 108.6), C–5′ (*δ*_C_ 116.2), C–6′ (*δ*_C_ 154.6), and C–7′ (*δ*_C_ 174.1) confirmed the second pentyl group at C–6′. Furthermore, the methoxy group (*δ*_H_ 3.84) showed an HMBC correlation with C–4 (*δ*_C_ 164.9), indicating that it was located at C–4 (Figures S17–S19).

### 2.2. Antimicrobial Activity

#### 2.2.1. Zone of Inhibition Test (ZOI) Disk Diffusion Method

The antimicrobial activity of the *C. skottsbergii* extract was assessed using the Kirby–Bauer disk diffusion method to ensure reproducibility and relatively rapid screening capability [29]. Results revealed significant antimicrobial activity of the extract, with the ZOI ranging from 14 to 25 mm (Table 2) against the tested strains. The positive control, ampicillin (10 µg per disk), exhibited ZOI values ranging from 20 to 38 mm. The extract displayed the highest antimicrobial activity against the Gram-negative bacterium *E. coli* compared to the Gram-positive bacteria MRSA USA-300 LAC. DMSO served as the negative control with no antibacterial properties.

For MSSA 8325-4, a ZOI of 25 mm for ampicillin and 16 mm for the *C. skottsbergii* extract (Figure S21) suggests that both samples are effective against this strain, indicating likely susceptibility.

#### 2.2.2. Minimal Inhibitory Concentration (MIC) Broth Microdilution Method

To evaluate their antimicrobial potential, extracts and the three isolated compounds from *C. skottsbergii*, namely clarosione (**1**), (*S*)-usnic acid (**2**), and perlatolic acid (**3**), were tested against both Gram-positive and Gram-negative strains. These strains included methicillin-resistant *S. aureus* (MRSA), methicillin-sensitive *S. aureus* (MSSA), *E. coli*, *Klebsiella pneumoniae*, and *Pseudomonas aeruginosa*. The MICs of the *C. skottsbergii* compounds and the positive controls, vancomycin and gentamicin, were also determined (Table 3).

Clarosione (**1**) exhibited weak antimicrobial activity against MRSA USA-300, MSSARI13, MSSA 8325-4, MSSA Newman, *E. coli*, *K. pneumoniae*, and *P. aeruginosa*, with MICs ranging from 32 to 128 µg/mL. Notably, clarosione (**1**) showed greater inhibitory activity against Gram-negative bacteria compared to (*S*)-usnic acid (**2**) and perlatolic acid (**3**).

The literature reports that (+)-usnic acid is effective in vitro against *Staphylococcus epidermidis*, *S. aureus*, and *S. haemolyticus*, with MICs of 3.12, 12.5, and 12.5 µg/mL, respectively [30]. Consistent with these findings, (*S*)-usnic acid (**2**) showed moderate activity against most *S. aureus* strains in our study, with MIC values of 2–8 µg/mL. MRSA HBP10 was particularly susceptible (MIC = 2 µg/mL), while MRSA USA-300 LAC, MSSA RI13, and MSSA LUU7, displayed MICs of 4–8 µg/mL.

Perlatolic acid (**3**) exhibited greater antimicrobial potency than (*S*)-usnic acid (**2**), with MIC values of 1–2 µg/mL for most tested strains, including MRSA HBP10 (1 µg/mL) and MRSA USA-300 (2 µg/mL). In contrast, MSSA LUU7 showed higher resistance to perlatolic acid, with a MIC of 8 µg/mL. Overall, perlatolic acid (**3**) demonstrated superior antimicrobial activity compared to (*S*)-usnic acid (**2**), particularly against MRSA and certain MSSA strains.

#### 2.2.3. Time–Kill Kinetics Assay

**Figure 6:**
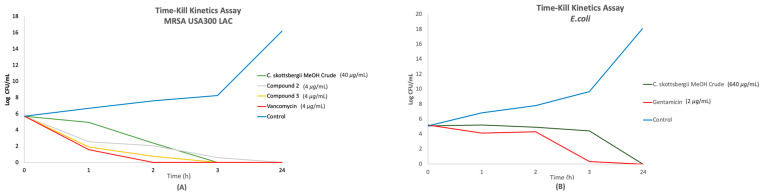
Time–kill kinetics assay of compounds **2** and **3**, *C. skottsbergii* methanol crude extract, and vancomycin used as a positive control against MRSA USA 300 LAC (**A**), and *C. skottsbergii* methanol crude extract and gentamicin used as a positive control against *E. coli* (**B**).

A time–kill study was conducted to assess the antimicrobial activity of compounds **2** and **3** and a methanol crude extract of *C. skottsbergii*, against MRSA USA300 LAC and *E. coli* (Figure 6). For MRSA USA300 LAC (Figure 6A), compound **3** (4 μg/mL) and the methanol crude extract (40 μg/mL) demonstrated strong killing effects within 3 h, similar to that of the positive control, vancomycin (4 μg/mL), which killed within 2 h. In contrast, compound **2** (4 μg/mL) acted more slowly, achieving complete killing after 24 h. Regarding *E. coli* (Figure 6B), the methanol crude extract (640 μg/mL) displayed a gradual killing effect over 24 h, whereas gentamicin (2 μg/mL) killed *E. coli* within 3 h. Overall, these results illustrate a rapid antibacterial effect for several agents against MRSA, while activity against *E. coli* was slower, except for gentamicin.

### 2.3. Sulforhodamine B (SRB) Cytotoxicity Assay

**Figure 7:**
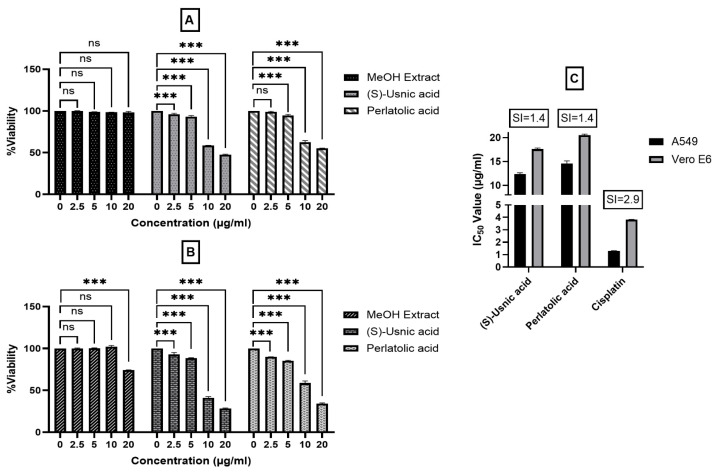
Cytotoxicity of the MeOH extract, (*S*)-usnic acid (**2**), and perlatolic acid (**3**) as measured by SRB assay after 72 h of exposure. Viability of Vero E6 (non-tumorigenic) cells (**A**) and A549 (non-small lung cancer) cells (**B**) was measured across increasing concentrations. (**C**) IC_50_ values of and selectivity indices (SI) for (*S*)-usnic acid (**2**), perlatolic acid (**3**), and cisplatin against A549 cells and Vero E6 cells. The study indicates that (*S*)-usnic acid (**2**) and perlatolic acid (**3**) produce significant, dose-dependent cytotoxic effects on A549 lung cancer cells, which are far more sensitive than Vero E6 normal cells. In contrast, the MeOH extract shows minimal cytotoxicity. Notably, (*S*)-usnic acid (**2**) and perlatolic acid (**3**) reduce cell viability significantly at concentrations above 5 µg/mL. With IC_50_ values revealing higher sensitivity in A549 cells, both compounds displayed selectivity indices (SI) of 1.4, indicating a modest selectivity toward cancer cells, although this is lower than cisplatin (SI = 2.9). ns = not significant, *** *p* < 0.001.

Compounds **2** and **3** and a methanol (MeOH) extract were evaluated for their cytotoxicity against human non-small cell lung cancer A549 and Vero E6 cells using an SRB assay (Figure 7).

## 3. Materials and Methods

### 3.1. General Experimental Procedures

Ultraviolet (UV) spectra were recorded on a Shimadzu PharmaSpec-1800 UV–visible spectrophotometer (Shimadzu Scientific Instruments, Columbia, MD, USA). Infrared (IR) radiation spectra were measured using a Thermo Scientific Nicolet iS 10 FTIR spectrometer. Optical rotation and the [*α*]_D_ value was determined using the Autopol^®^ IV Automatic Polarimeter (Rudolph Research Analytical, Hackettstown, NJ, USA). X-ray crystallographic data were collected at 298 K on a Bruker D8 VENTURE four-circle κ-geometry diffractometer. The instrument was equipped with an Incoatec IμS 3.0 microfocus sealed tube (Cu Kα radiation; λ = 1.54178 Å), a multilayer mirror monochromator, and a Photon III M14 area detector. One-dimensional (1D) and two-dimensional (2D) nuclear magnetic resonance (NMR) spectra were acquired on a Bruker AVANCE DRX-400 and a Bruker AV-600 NMR spectrometer (Bruker, Billerica, MA, USA). Chemical shifts (*δ*) are reported in parts per million using tetramethylsilane (TMS) as the internal standard. Data were processed using the MestReNova version 14.2.1-27684 software with the residual solvent signals of CDCl_3_ (*δ*_H_ 7.26, *δ*_C_ 77.16) and MeOD (*δ*_H_ 3.31, *δ*_C_ 49.0) as reference standards. All fractions were monitored by thin-layer chromatography (TLC) using silica gel 60 F254 aluminum plates (0.25 mm, Merck, Darmstadt, Germany). Spots were visualized by UV detection at 254 and 365 nm and by subsequent heating after soaking the plates with *p*-anisaldehyde solution. For large-scale separation, column chromatography (CC) utilized silica gel (230–400 mesh and 480–800 mesh, Sorbent Technologies, Atlanta, GA, USA) and Sephadex LH-20 (GE Healthcare, Piscataway, NJ, USA) as stationary phases. Preparative HPLC was conducted using a Thermo Scientific Ultimate 3000 system equipped with a photodiode array detector. Separations were achieved on an ALLTECH Econosil reversed-phase C_18_ column (10 µm, 10 × 250 mm, Lot No. 3513, Part No.6231, Serial No. 605080632.1) with a flow rate of 2.0 mL/min. The corresponding lab received a Biosafety Level 2 permit from the University of Hawaii Institutional Biosafety Committee (IBC), protocol number: B24-101424, expiration date: 30 September 2027, Biosafety level and Risk Group: BSL2 (Biosafety Level 2).

### 3.2. Lichen Materials

*C. skottsbergii* was collected in the Lotus Buddhist Monastery at Mountain View, Big Island, Hawai’i, USA, in February 2023. Identification was performed by Dr. Clifford Smith, and a voucher specimen (BISH 800047) was deposited at the Bernice Pauahi Bishop Herbarium.

### 3.3. Extraction and Isolation

The clean and air-dried Hawaiian lichen *C. skottsbergii* (139.53 g) was ground into fine powder using a blender to disrupt the cell walls and facilitate extraction. The powdered lichen was extracted with methanol (3 × 470 mL) at room temperature for three days. After filtering and concentrating the extract under reduced pressure, 3.8 g of methanol crude extract was obtained. This crude extract (10.8 g) was washed with MeOH to give two main fractions: mother liquor (ML) and yellow powder (YP). The main fraction ML (4.2 g) was first subjected to Sephadex LH-20 column chromatography (CC) using 100% MeOH as the mobile phase, followed by CC on silica gel using a gradient of *n*-hexane–DCM–MeOH, yielding compounds **2** (5.2 mg) and **3** (24.2 mg). Fraction YP (6.4 g) was separated on a RP18 column chromatography gradient of MeOH–H_2_O-acetone to give compound **2** (178.4 mg) and sub-fraction YPA (6.4 mg). The YPA (6.4 mg) was purified by a semi-preparative HPLC reversed-phase C_18_ column (MeCN–H_2_O, 70:30 to 90:10, flow rate 1.7 mL/min) to yield compound **1** (*t*_R_ 25.2, 0.9 mg) (Figure S20).

*Clarosione* (**1**): yellow needle, m.p. 191–192 °C; UV (CHCl_3_) *λ*_max_ (log *ε*) 210 (3.77), 280 (2.91) nm; IR ν_max_ 1690 (C=O), 1633 (C=C) cm^−1^; ^1^H, ^13^C, and 2D-NMR data, see Table 1; HRESIMS *m*/*z* 358.9635 [M + H]^+^ (calcd. for C_15_H_10_Cl_3_O_4_, *m*/*z* 358.9639).

### 3.4. Single-Crystal X-Ray Structure Determination of Clarosione (**1**)

Crystals of clarosione (**1**) (Figure 4) were obtained from hexane/dichloromethane. A yellow needle of approximate dimensions 0.060 mm × 0.060 mm × 0.200 mm was used in the X-ray diffraction experiment. Data were collected at 298 K on a Bruker D8 VENTURE four-circle κ-geometry diffractometer equipped with an Incoatec IμS 3.0 microfocus sealed tube with a multilayer mirror monochrometer and a Photon III M14 area detector; Cu Kα radiation (*λ* = 1.54178 Å) was used. Data were collected to a maximum θ angle of 77.27° (0.79 Å resolution) and integrated using the program SAINT, part of the Bruker APEX5 software package, yielding 37370 reflections, of which 3087 were independent (average redundancy 12.11; completeness = 99.8%, R_int_ = 8.27%, R_sig_ = 3.63%) and 2788 (90.31%) were greater than 2σ(F^2^)). Data were corrected for absorption effects (SADABS), with T_min_ = 0.5973 and T_max_ = 0.7541. The final cell constants are based upon 8917 reflections above 20σ(I) with 10.56° < 2θ < 150.1°. Crystal data for clarosione: C_15_H_9_Cl_3_O_4_; M = 359.57 g/mol; orthorhombic space group P2_1_2_1_2_1_ (no. 19); a = 4.37920(10) Å; b = 17.3020(3) Å; c = 19.1464(3) Å; V= 1450.70(5) Å^3^; Z = 4; (Z’ = 1); T = 298 K; μ(Cu Kα) = 5.870 mm^−1^; and d_calc_ = 1.646 g/cm^3^. The structure was solved using the program SHELXT, part of the Bruker APEX5 software package, and refined using SHELXL. All non-H atoms were refined with anisotropic displacement parameters; and all H atoms were refined with isotropic displacement parameters. Some of their coordinates were refined freely and some on calculated positions using a riding model with their U_iso_ values constrained to 1.5 times the U_eq_ of their pivot atoms for terminal sp^3^ C-atoms and 1.2 times for all other C-atoms. In total, 1247 Friedel/Bijvoet pairs out of a possible 1254 (99.4% coverage) were collected. The Flack x value for the absolute structure of the crystal was 0.051(9), determined using 1044 selected quotients (Parsons’ method; SHELXL). The Hooft Student’s *t*-test value was (PLATON) y = 0.040(8), with P2(true) = 1.000. The final full-matrix least-squares refinement on F^2^ with 207 variables converged at R1 = 3.30% for the observed data and wR2 = 8.53% for all data; the goodness-of-fit (S) was 1.064. The largest peak in the final difference electron density synthesis was 0.304 e-/Å^3^ and the largest hole was −0.326 e-/Å^3^ with an RMS deviation of 0.044 e-/Å^3^. A Mogul Geometry Check (CCDC/Mercury) of all bond lengths and angles revealed no unusual values. CCDC 2419297 and 2389503 contains the supplementary crystallographic data for (**1**) and (**2**). These data can be obtained free of charge via: https://www.ccdc.cam.ac.uk/ (or from the CCDC, 12 Union Road, Cambridge CB2 1EZ, UK; Fax: +44-1223-336033; E-mail: deposit@ccdc.cam.ac.uk). The supplementary tables of data for compound **1** are found in Tables S1–S10.

### 3.5. Single-Crystal X-Ray Structure Determination of (S)-Usnic Acid (**2**) [31]

Crystals of (*S*)-usnic acid (**2**), as shown in Figure 4, were obtained using methanol as the solvent. A greenish-yellow crystal of approximate dimensions 0.080, 0.080, and 0.120 mm was used in the X-ray diffraction experiment. Data were collected at 298 K on a Bruker D8 VENTURE four-circle κ-geometry diffractometer equipped with an Incoatec IμS 3.0 microfocus sealed tube with a multilayer mirror monochromator and a Photon III M14 area detector. Cu Kα radiation was use (*λ* = 1.54178 Å). Data were collected to a maximum θ angle of 77.65° (0.79 Å resolution) and integrated using the program SAINT, part of the Bruker APEX5 software package, yielding 76769 reflections, of which 6638 were unique (average redundancy 11.57, completeness = 99.5%, R_int_ = 5.25%, R_sig_ = 2.15%) and 6273 (94.50%) were greater than 2σ(F^2^)). Data were corrected for absorption effects (SADABS), T_min_ = 0.691 and T_max_ = 0.754. The final unit cell constants are based upon 9022 reflections above 20σ(I) with 6.354 < 2θ < 154.8°. Crystal data for (*S*)-usnic acid: C_18_H_16_O_7_; M = 344.31 g/mol; orthorhombic space group P2_1_2_1_2_1_ (no. 19); a = 8.06570(10) Å; b = 19.0635(3) Å; c = 20.3423(3) Å; V = 3127.84(8) Å^3^; Z = 8 (Z′ = 2); T = 298 K; μ (Cu Kα) = 0.961 mm^−1^; and d_calc_ = 1.462 g/cm^3^. The structure was solved using the program SHELXT, part of the Bruker APEX5 Software Package. All non-H atoms were refined anisotropically, and 26 out of 32 H atoms were refined with isotropic displacement parameters at idealized positions using a riding model, while the other six hydroxy H’s were refined freely (*vide infra*). In total, 2891 Friedel/Bijvoet pairs out of a possible 2904 (99.6% coverage) were collected. The Flack x value was 0.02(4), determined using 2643 selected quotients (Parsons’ method; SHELXL). The Hooft Student’s *t*-test value was (PLATON) y = 0.04(3), WITH P2 (true) = 1.000. The final full-matrix least-squares refinement on F^2^ with 485 variables converged at R1 = 2.68% for the observed data and wR2 = 7.69% for all data; the goodness-of-fit (*S*) was 1.032. The largest peak in the final difference electron density synthesis was 0.167 e-/Å^3^ and the largest hole was 0.108 e-/Å^3^ with an RMS deviation of 0.027 e-/Å^3^.

**Figure 8:**
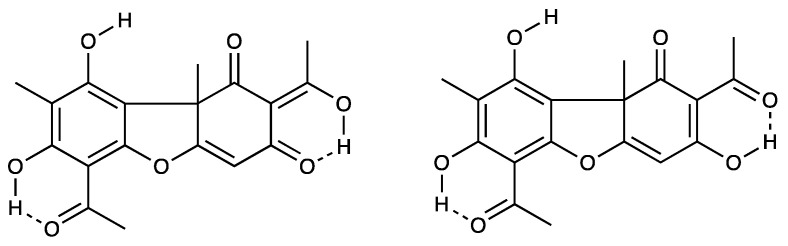
Isoenergetic tautomers of the structure of usnic acid.

The structural results of (**2**) reveal strong, nearly symmetrical intramolecular O3-H3···O2 and O10-H10···O9 hydrogen bonds with donor–acceptor distances of 2.406(2) and 2.415(2) Å, respectively, whereas the O6-H6···O5, O7-H7···O1 and O13-H13···O12, O14-H14···O8 intramolecular hydrogen-bonding interactions are weaker and asymmetrical (Figure 8). In toto, the observed molecular geometry is consistent with a structure that is a weighted average of the two nearly isoenergetic tautomers shown below (Figure 8) [26]. The supplementary tables of data for compound **2** are found in Tables S11–S20.

Note: the X-ray structure of (*R*)-usnic acid has been reported [32]; however, the two hydrogen atoms involved in the symmetrical O-H···O hydrogen bonds were apparently not located, and their fractional coordinates were not reported (CSD refcode: XIFWOZ; CCDC 2389503).

### 3.6. Bacterial Strains

The study utilized the following bacterial strains from the American Type Culture Collection (ATCC, Manassas, VA, USA): Gram-negative strains *E. coli* 9637, *K. pneumoniae* NCTC 9633, and *P. aeruginosa* PA01; and Gram-positive strains methicillin-resistant *S. aureus* (MRSA USA-300 LAC) and methicillin-susceptible *S. aureus* (MSSA 8325-4 and MSSA Newman); the Hawaiian bacteria strains MRSA POB2, MRSA HBP10, MRSA KEH 19.2, MSSA RI13, MSSA LUU7, MSSA ONE6, and MSSA RI27 were obtained from Dr. Tracy Wiegner, the Marine Science Department at UH Hilo [4]. All strains were grown on Mueller–Hinton (MH) agar plates and Mueller–Hinton broth (Becton, Dickinson and Company, Franklin Lakes, NJ, USA). The Hawaiian MSSA and MRSA strains were isolated from various locations on the Island of Hawaii, including Pohoiki Beach Park (POB2), Honoli’i Beach Park (HBP10), Richardson’s Beach Park—Back (RI27), Richardson’s Beach Park (RI13), Kehena Beach (KEH 19.2), Onekahakaha Beach Park (ONE6), and Wailuku River (LUU7). These strains contained antimicrobial resistance genes such as *blaZ* (*β*-lactam resistance) in MSSA and the mecA gene, erm(C), mph(C), and msr(A) in MRSA [4].

### 3.7. Antimicrobial Assay: Disk Diffusion Method

The antimicrobial susceptibility of crude extracts and samples from *C. skottsbergii* was initially assessed using the disk diffusion method against MRSA USA-300 LAC, *E. coli*, and MSSA 8325-4 [33]. Tested culture strains were grown overnight in MH broth at 35–37 °C. After incubation, each strain was diluted in sterile MH broth and adjusted to a turbidity of the 0.5 McFarland standard (1.5 × 10^8^ colony-forming units/mL) before use. Then, 40 µL of the adjusted bacteria strain was evenly spread onto each plate using sterile cotton. The concentration of each lichen’s sample (4.0 mg/mL) was prepared and dissolved in dimethyl sulfoxide (DMSO, Sigma-Aldrich, Saint Louis, MO, USA). Then, 20 µL of the sample (80 µg) was pipetted onto disks, aseptically transferred onto MH agar plates, and incubated at 35–37 °C for 24 h. Ampicillin (10 µg, Oxoid, Basingstoke, Hampshire RG24 8PW, UK) was used as a positive control and DMSO as a solvent control in the disk diffusion assay. Antimicrobial activity was assessed by recording zones of inhibition (ZOIs) in millimeters and categorizing the results according to clinical breakpoints and antibiotic dosing standards recommended by the European Committee on Antimicrobial Susceptibility Testing (EUCAST) and the European Society for Clinical Microbiology and Infectious Diseases (ESCMID). Ampicillin 10 µg against Enterobacterales was classified as resistant (R) ≤14 and susceptible (S) ≥14 (EUCAST breakpoint, 2023). Ampicillin is considered resistant to MRSA; therefore, no breakpoints exist according to EUCAST guidelines.

### 3.8. Determination of Minimum Inhibitory Concentrations (MIC)

This method was modified using a previous microdilution method [34]. In brief, samples (DMSO) were diluted in two-fold serial dilutions (128, 64, 32, 16, 8, 4, 2, 1, 0.5, and 0.25 µg/mL) with Mueller Hinton broth (MHB) in a 96-well plate. Next, 50 µL of bacteria was added to each well containing 5 × 10^4^ colony-forming units and incubated at 35–37 °C for 18–24 h. Next, 0.018% resazurin (15 µL) was added and incubated for 1–2 h; the corresponding color changes were used to determine the MIC values (µg/mL). Wells with bacterial growth remained pink, while those that turned blue indicated growth inhibition. The MIC was defined as the well with the lowest concentration of compounds where no visible bacterial growth was observed [35]. All experiments were performed in triplicate using vancomycin and gentamicin.

### 3.9. Time–Kill Kinetics Assay

The study examined the time–kill kinetics of compounds **2** and **3** and the methanol crude extract of *C. skottsbergii* against two different strains of bacteria: MRSA USA 300 LAC (Gram-positive) and *E. coli* (Gram-negative). Each strain (10^5^ CFU/mL) was inoculated into culture tubes with compound **2** or **3** at 4 μg/mL, or the methanol extract of *C. skottsbergii* at 40 μg/mL for MRSA USA 300 LAC and 640 μg/mL for *E. coli* in nutrient broth. A growth control without extract or compounds was included. Tubes were incubated at 37 °C. Bacteria were counted at 0, 1, 2, 3, and 24 h using the drop-plate method [36]. At each interval, a 10-fold serial dilution was performed to determine bacterial counts. Bactericidal activity was defined as at least a 99.9% reduction or a decrease of 3 log10 CFU/mL compared to control [37,38]. Each concentration was tested in triplicate.

### 3.10. Cell Lines

A549 human lung carcinoma epithelial cells (CCL-185^TM^) and Vero E6 (CRL-1586, African green monkey kidney cells) were sourced from the American Type Culture Collection (ATCC, Manassas, VA, USA). The cells were maintained in Dulbecco’s modified Eagle’s medium supplemented with heat-inactivated fetal bovine serum (FBS, 10%) and antibiotics (penicillin 1000 IU/mL and streptomycin 1000 μg/mL) at 37 °C in a humidified incubator with 5% CO_2_.

### 3.11. Sulforhodamine B (SRB) Cytotoxicity Assay

Cytotoxicity was assessed using the sulforhodamine B (SRB) assay, following established protocols [39]. A549 (6000 cells/well) or Vero E6 (8000 cells/well) cells were seeded in 96-well plates and incubated for 24 h. Cells were incubated for 72 h with increasing concentrations of the test sample (1.25–50 μg/mL), 0.5% DMSO as the negative control, and 0.5–20 μM cisplatin as a positive control. After treatment, cells were fixed with 10% trichloroacetic acid, washed, and stained with 0.4% SRB in 1% acetic acid. Unbound dye was removed by washing with 1% acetic acid. Protein-bound dye was solubilized using a 10 mM Tris buffer at pH 10.0, after which optical density was measured at 515 nm. All experiments were performed in triplicate. Percent cell death was calculated using the provided equation:

$$\%\;Cell\;death=\frac{\left(ODc\;-\;{OD}_{0})\;-\;(ODs\;-\;{OD}_{0}\right)}{\left(ODc\;-\;{OD}_{0}\right)}\times 100$$

OD_0_ denotes the optical density of cells measured prior to the addition of the test sample/standard on day 0, serving as the baseline measurement. OD_C_ indicates the optical density in control wells at 72 h in the absence of the test sample, providing a baseline measurement. OD_S_ indicates the optical density of cells after 72 h of incubation with the test sample or standard compound, allowing comparison to ODc to assess the sample’s effect.

The median inhibitory concentration (IC_50_) is the concentration at which 50% cell death is observed. It was determined by plotting cell death percentage against the test sample concentration. Non-linear curve fitting was performed using GraphPad Prism software (GraphPad 10.0.2, San Diego, CA, USA). The selectivity index (SI) was calculated using the following equation: SI = IC_50_ Vero E6 cells/IC_50_ A549 cells [40,41,42,43,44,45,46,47,48,49,50,51,52,53,54].

### 3.12. Statistical Analysis

Data are expressed as mean ± standard error of the mean (SEM) from three independent experiments. For statistical analysis, Student’s *t*-test or ANOVA with Tukey’s test was used. Significance is denoted as * *p* < 0.05, ** *p* < 0.01, and *** *p* < 0.001. The IC_50_ values for A549 cells were calculated by non-linear curve fitting in Prism software (GraphPad 10.0.2, San Diego, CA, USA), ensuring a goodness-of-fit with *R*^2^ > 0.9 and *p* > 0.5 (runs test).

## 4. Conclusions

This study reports the discovery of a new and rare compound, clarosione (**1**), from the lichen *C. kottsbergii,* along with the first description of its X-ray structure. Additional compounds isolated from *C. skottsbergii*, specifically (*S*)-usnic acid (**2**) and perlatolic acid (**3**), demonstrate significant antimicrobial activity against Gram-positive bacteria, including multidrug-resistant MRSA and MSSA strains obtained from Hawaii. These results underscore the potential of *C. skottsbergii*-derived natural products and support their further development for the treatment of resistant Gram-positive infections. However, their limited efficacy against Gram-negative bacteria indicates the need for structural modification or combination with other agents to enhance their broad-spectrum activity. Given the increasing prevalence of antibiotic-resistant pathogens, particularly MRSA, these natural compounds offer a promising avenue for the development of antimicrobials. Further research is necessary to characterize, optimize, and evaluate the clinical potential of these compounds, particularly in regions such as Hawaii, where resistant infections pose a significant burden.

## Figures and Tables

**Table 1:** ^1^H (600 MHz) and ^13^C NMR (150 MHz) data in CDCl_3_ of clarosione (**1**).

Position	*δ*_C_	*δ*_H_ [Mult, *J* in Hz]	HMBC (^1^H ⟶ ^13^C)
1	155.3	-	-
2	116.4	-	-
3	144.7	-	-
4	112.2	-	-
5	145.2	-	-
6	135.5	-	-
7	126.1	7.44 (d, 8.7)	C–5, C–6, C–8, C–13
8	120.8	7.94 (d, 8.7)	C–9, C–12
9	180.7	-	-
10	107.4	-	-
11	149.2	-	-
12	150.1	-	-
13	119.9	-	-
1-OH	-	13.08 (s)	C–1, C–2, C–9, C–10, C–11
3-CH_3_	19.0	2.67 (s)	C–2, C–3, C–4
5-OCH_3_	61.7	4.20 (s)	C–5

**Table 2:** Antimicrobial activities of samples from *C. skottsbergii* extract using disk diffusion assay.

Bacteria	Inhibition Zone (mm)		
	*C. skottsbergii* Methanol Extract (4 mg/mL), 80 (µg/disk)	Negative Control (DMSO)	Positive Control Ampicillin (10 µg/disk)
*E. coli*	25	0	20
MRSA USA-300 LAC	14	0	38
MSSA 8325-4	16	0	25

**Table 3:** Antimicrobial activity assessed by the broth microdilution method.

Bacteria	MIC (µg/mL)					
	Clarosione (1)	(*S*)-Usnic Acid (2)	Perlatolic Acid (3)	Crude Extract	Vancomycin	Gentamicin
MRSA POB2	nt	4	2	40	0.5	-
MRSA USA-300	128	4	2	40	0.5	-
MRSA HBP10	nt	2	1	20	1	-
MSSA RI13	128	4	2	40	0.5	-
MSSA LUU7	nt	4	8	80	0.5	-
MRSA KEH 19.2	nt	8	2	40	0.5	-
MSSA ONE6	nt	4	2	40	0.5	-
MSSA RI27	nt	4	2	40	1	-
MSSA 8325-4	32	4	2	40	0.5	-
MSSA Newman	32	4	4	40	0.5	-
*E. coli*	128	128	128	640	-	0.25
*K. pneumoniae*	32	128	128	640	-	0.25
*P. aeruginosa*	64	128	128	640	-	0.25

nt = not test.

## Data Availability

The original contributions presented in this study are included in the article/Supplementary Material. Further inquiries can be directed to the corresponding author.

## Supplementary Materials

The following supporting information can be downloaded at: https://www.mdpi.com/article/10.3390/ph19010174/s1, NMR spectroscopy. Figure S1: ^1^H NMR spectrum of clarosione (**1**); Figure S2: ^13^C NMR spectrum of clarosione (**1**); Figure S3: COSY spectrum of clarosione (**1**), Figure S4: HSQC spectrum of clarosione (**1**); Figure S5: HMBC spectrum of clarosione (**1**); Figure S6: HRESIMS spectrum of clarosione (**1**); Figure S7: IR radiation spectrum of clarosione (**1**); Figure S8:UV spectrum of clarosione (**1**); Figure S9: ^1^H NMR spectrum of (*S*)-usnic acid (**2**); Figure S10: ^13^C NMR spectrum of (*S*)-usnic acid (**2**); Figure S11: COSY spectrum (CDCl_3_) of (*S*)-usnic acid (**2**); Figure S12: HSQC spectrum of (*S*)-usnic acid (**2**); Figure S13: HMBC spectrum of (*S*)-usnic acid (**2**); Figure S14: NOESY spectrum of (*S*)-usnic acid (**2**); Figure S15: ^1^H NMR spectrum of perlatolic acid (**3**); Figure S16: ^13^C NMR spectrum of perlatolic acid (**3**); Figure S17: COSY spectrum of perlatolic acid (**3**); Figure S18: HSQC spectrum of perlatolic acid (**3**); Figure S19: HMBC spectrum of perlatolic acid (**3**); Figure S20: RP–High-performance liquid chromatograms of clarosione (**1**) *t*_R_ 25.2, 0.9 mg, MeCN–H_2_O, 70:30 to 90:10, flow rate 1.7 mL/min; Figure S21: *C. skottsbergii* extract using disk diffusion assay; Table S1: Data collection details for clarosione (**1**); Table S2: Sample and crystal data for clarosione (**1**); Table S3: Data collection and structure refinement for clarosione (**1**); Table S4: Atomic coordinates and equivalent isotropic atomic displacement parameters (Å^2^) for clarosione (**1**); Table S5: Bond lengths (Å) for clarosione (**1**); Table S6: Bond angles (°) for clarosione (**1**); Table S7. Torsion angles (°) for clarosione (**1**); Table S8: Anisotropic atomic displacement parameters (Å^2^) for clarosione (**1**); Table S9: Hydrogen atomic coordinates and isotropic atomic displacement parameters (Å^2^) for clarosione (**1**); Table S10: Hydrogen bond distances (Å) and angles (°) for clarosione (**1**); Table S11: Data collection details for (*S*)-usnic acid (**2**); Table S12: Sample and crystal data for (*S*)-usnic acid (**2**); Table S13: Data collection and structure refinement for (*S*)-usnic acid (**2**); Table S14: Atomic coordinates and equivalent isotropic atomic displacement parameters (Å^2^) for (*S*)-usnic acid (**2**); Table S15: Bond lengths (Å) for (*S*)-usnic acid (**2**); Table S16: Bond angles (°) for (*S*)-usnic acid (**2**); Table S17: Torsion angles (°) for (*S*)-usnic acid (**2**); Table S18: Anisotropic atomic displacement parameters (Å^2^) for (*S*)-usnic acid (**2**); Table S19: Hydrogen atomic coordinates and isotropic atomic displacement parameters (Å^2^) for (*S*)-usnic acid (**2**); Table S20: Hydrogen bond distances (Å) and angles (°) for (*S*)-usnic acid (**2**).

## Abbreviations

The following abbreviations are used in this manuscript:

MDR	multidrug-resistance
MRSA	methicillin-resistant *S. aureus*
MSSA	methicillin-susceptible *S. aureus*
MIC	Minimal Inhibitory Concentration
ZOI	Zone of Inhibition
EUCAST	European Committee on Antimicrobial Susceptibility Testing
SRB	Sulforhodamine B Cytotoxicity Assay
MeOH	Methanol
DCM	Dichloromethane
NMR	Nuclear Magnetic Resonance spectroscopy
