# Supplementary material for: A New Trichlorinated Xanthone and Compounds Isolated from Cladonia skottsbergii with Antimicrobial Properties
Source: Pharmaceuticals (Basel). 2026 Jan 19;19(1):174. doi: 10.3390/ph19010174 (PMC12844737; doi:10.3390/ph19010174)
Supplement: Supplementary file 1 [file pharmaceuticals-19-00174-s001.zip › pharmaceuticals-4058862-supplementary.pdf]

SUPPLEMENTARY DATA

A New Trichlorinated Xanthone and Isolated Compounds Isolated from *Cladonia skottsbergii* with Antimicrobial Properties

Marvin J. Rositzki [1,2], Achara Raksat [1], Charles J. Simmons [3], Clifford Smith [4], Reverend Danette V. Choi [5], Supakit Wongwiwatthananukit [2,6], Leng Chee Chang [1,*]

[1] Department of Pharmaceutical Sciences, The Daniel K. Inouye College of Pharmacy, University of Hawai'i at Hilo, Hilo, HI, 96720, USA; rositzki@hawaii.edu; achara@hawaii.edu; lengchee@hawaii.edu

[2] Department of Pharmacy Practice, The Daniel K. Inouye College of Pharmacy, University of Hawai'i at Hilo, Hilo, HI, 96720, USA; rositzki@hawaii.edu; supakit@hawaii.edu

[3] X-ray diffraction Laboratory, Department of Chemistry, 200 West Kawili Street, University of Hawai'i at Hilo, Hilo 96720, USA; simmonsc@hawaii.edu

[4] Department of Botany, University of Hawai'i at Manoa, Honolulu 96822, USA; cliff@hawaii.edu

[5] Priest (Monk) of Bang San Ho Temple Yun Hwa Denomination of World Social Buddhism dba Lotus Buddhist Monastery, Mountain View, HI, 96771, USA; manjin@yunhwasangha.org

[6] College of Pharmacy, Rangsit University, Pathum Thani 12000, Thailand; supakit.w@rsu.ac.th

[*] **Corresponding Author to**: lengchee@hawaii.edu; Tel.: +1-808-932-8124,

List of Contents

		page
**Figure S1.**	^{1}H NMR spectrum (600 MHz, $CDCl_3$) of clarosione (**1**)	3
**Figure S2.**	^{13}C NMR spectrum (150 MHz, $CDCl_3$) of clarosione (**1**)	3
**Figure S3.**	COSY spectrum ($CDCl_3$) of clarosione (**1**)	4
**Figure S4.**	HSQC spectrum ($CDCl_3$) of clarosione (**1**)	4
**Figure S5.**	HMBC spectrum ($CDCl_3$) of clarosione (**1**)	5
**Figure S6.**	HRESIMS spectrum of clarosione (**1**)	5
**Figure S7.**	IR radiation spectrum of clarosione (**1**)	6
**Figure S8.**	UV spectrum of clarosione (**1**)	6
**Figure S9.**	^{1}H NMR spectrum (400 MHz, $CDCl_3$) of (*S*)-usnic acid (**2**)	7
**Figure S10.**	^{13}C NMR spectrum (100 MHz, $CDCl_3$) of (*S*)-usnic acid (**2**)	7
**Figure S11.**	COSY spectrum ($CDCl_3$) of (*S*)-usnic acid (**2**)	8
**Figure S12.**	HSQC spectrum ($CDCl_3$) of (*S*)-usnic acid (**2**)	8
**Figure S13.**	HMBC spectrum ($CDCl_3$) of (*S*)-usnic acid (**2**)	9
**Figure S14.**	NOESY spectrum ($CDCl_3$) of (*S*)-usnic acid (**2**)	9
**Figure S15.**	^{1}H NMR spectrum (400 MHz, $CDCl_3$) of perlatolic acid (**3**)	10
**Figure S16.**	^{13}C NMR spectrum (100 MHz, $CDCl_3$) of perlatolic acid (**3**)	10
**Figure S17.**	COSY spectrum ($CDCl_3$) of perlatolic acid (**3**)	11
**Figure S18.**	HSQC spectrum ($CDCl_3$) of perlatolic acid (**3**)	11
**Figure S19.**	HMBC spectrum ($CDCl_3$) of perlatolic acid (**3**)	12
**Figure S20**	RP–High-performance liquid chromatograms of clarosione (**1**) t_R 25.2, 0.9 mg, MeCN−H_2O, 70:30 to 90:10, flow rate 1.7 mL/min.	12
**Figure S21**	*C. skottsbergii* extract using disk diffusion assay	13
**Table S1.**	Data collection details for clarosione (**1**)	14
**Table S2.**	Sample and crystal data for clarosione (**1**)	15
**Table S3.**	Data collection and structure refinement for clarosione (**1**)	15
**Table S4.**	Atomic coordinates and equivalent isotropic atomic displacement parameters ($Å^2$) for clarosione (**1**)	16
**Table S5.**	Bond lengths (Å) for clarosione (**1**)	17
**Table S6.**	Bond angles (°) for clarosione (**1**)	17
**Table S7.**	Torsion angles (°) for clarosione (**1**)	18
**Table S8.**	Anisotropic atomic displacement parameters ($Å^2$) for clarosione (**1**)	18
**Table S9.**	Hydrogen atomic coordinates and isotropic atomic displacement parameters ($Å^2$) for clarosione (**1**)	19
**Table S10**	Hydrogen bond distances (Å) and angles (°) for clarosione (**1**)	19
**Table S11**	Data collection details for (*S*)-usnic acid (**2**)	19
**Table S12**	Sample and crystal data for (S)-usnic acid (**2**)	21
**Table S13**	Data collection and structure refinement for (*S*)-usnic acid (**2**)	21
**Table S14**	Atomic coordinates and equivalent isotropic atomic displacement parameters ($Å^2$) for (*S*)-usnic acid (**2**)	22
**Table S15**	Bond lengths (Å) for (*S*)-usnic acid (**2**)	23
**Table S16**	Bond angles (°) for (*S*)-usnic acid (**2**)	23
**Table S17**	Torsion angles (°) for (*S*)-usnic acid (**2**)	25
**Table S18**	Anisotropic atomic displacement parameters ($Å^2$) for (*S*)-usnic acid (**2**)	27
**Table S19**	Hydrogen atomic coordinates and isotropic atomic displacement parameters ($Å^2$) for (*S*)-usnic acid (**2**)	28
**Table S20**	Hydrogen bond distances (Å) and angles (°) for (*S*)-usnic acid (**2**)	28

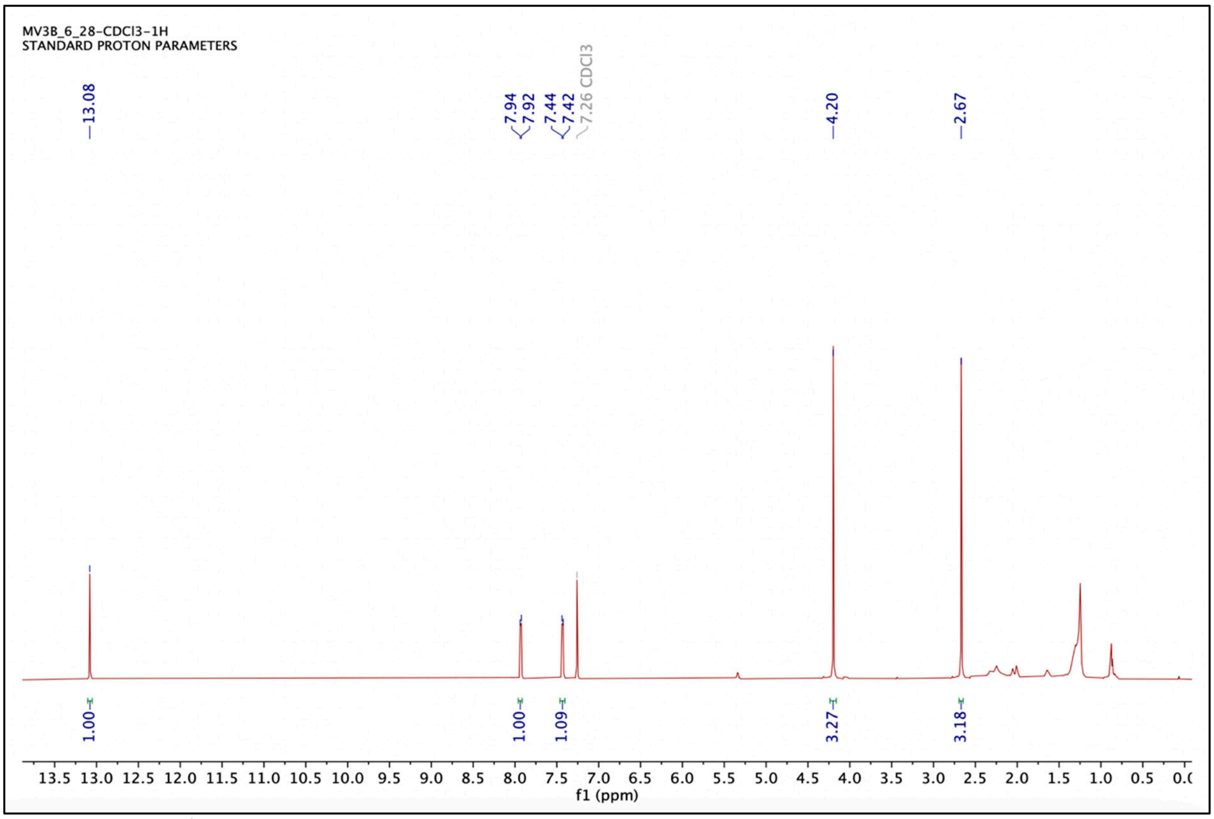

Figure S1. ^{1}H NMR spectrum (600 MHz, $CDCl_3$) of clarosione (**1**)

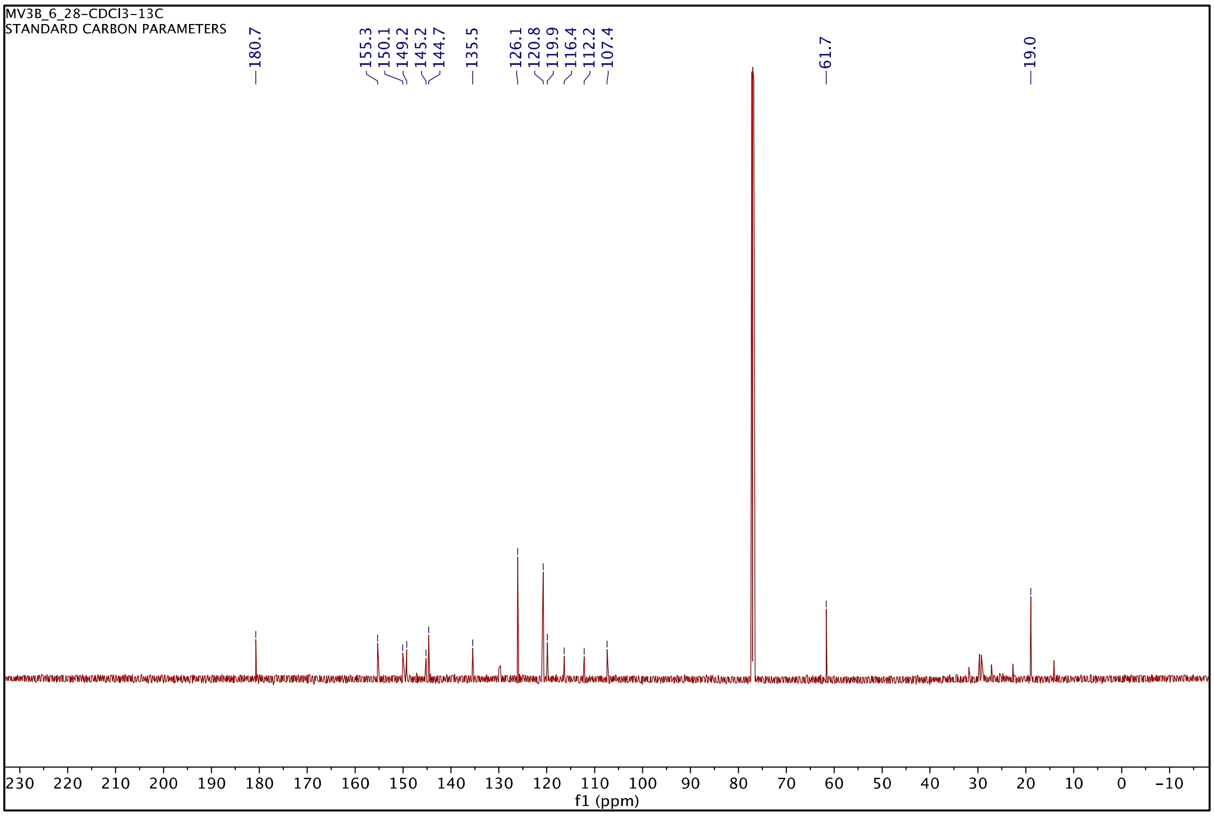

Figure S2. ^{13}C NMR spectrum (150 MHz, $CDCl_3$) of clarosione (**1**)

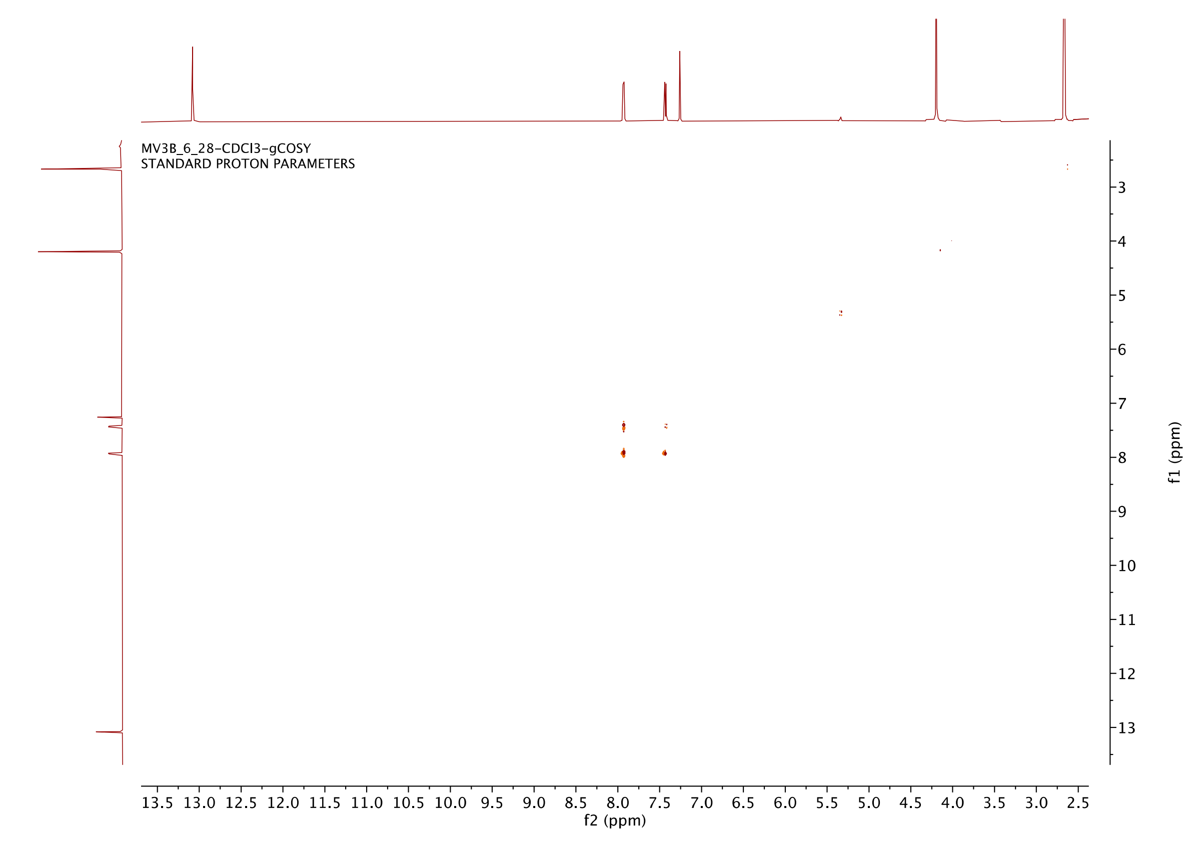

Figure S3. COSY spectrum ($CDCl_3$) of clarosione (**1**)

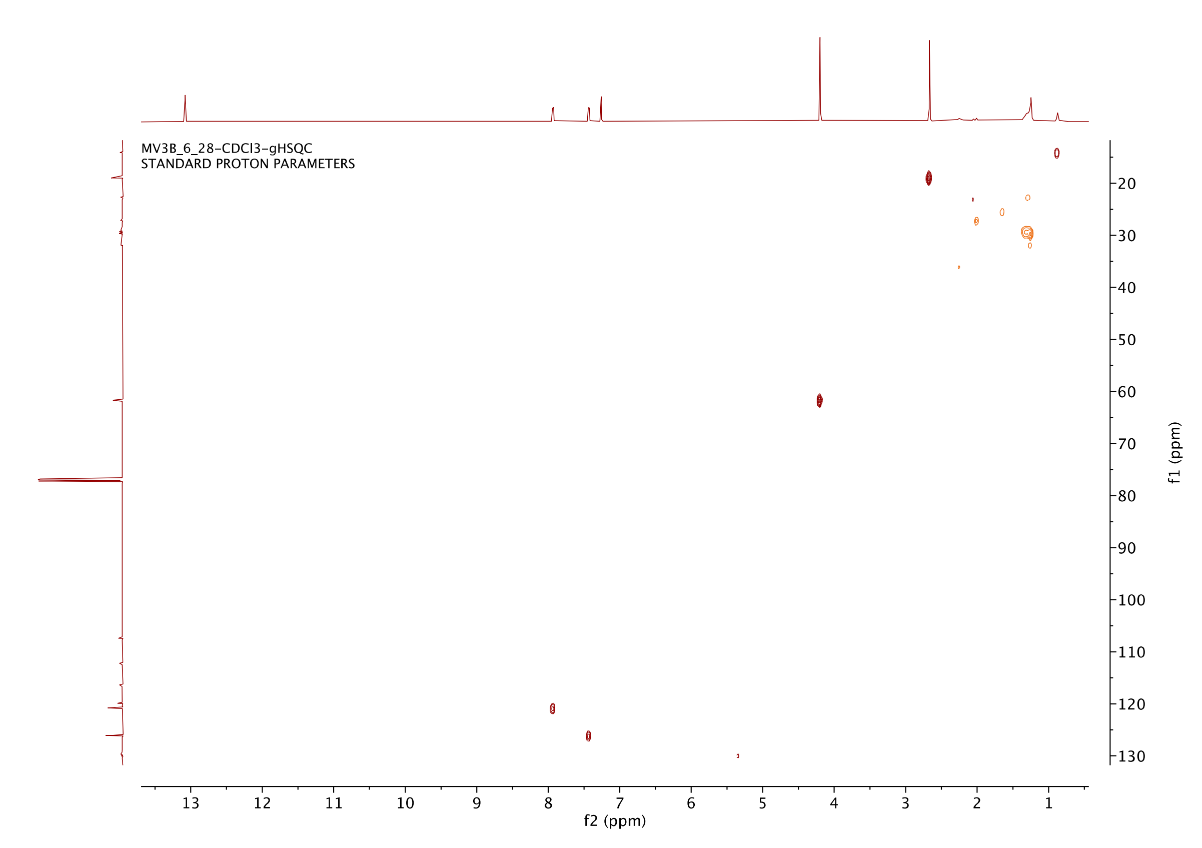

Figure S4. HSQC spectrum ($CDCl_3$) of clarosione (**1**)

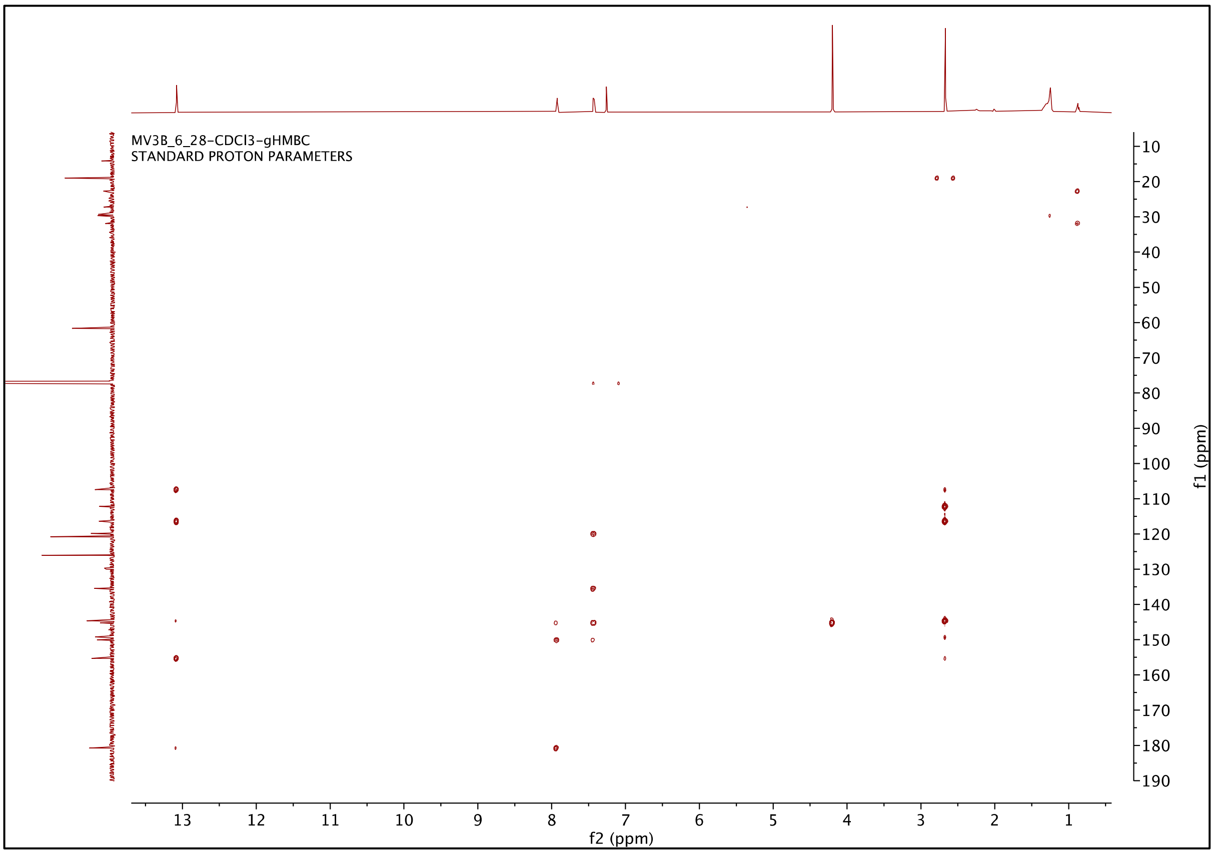

Figure S5. HMBC spectrum ($CDCl_3$) of clarosione (**1**)

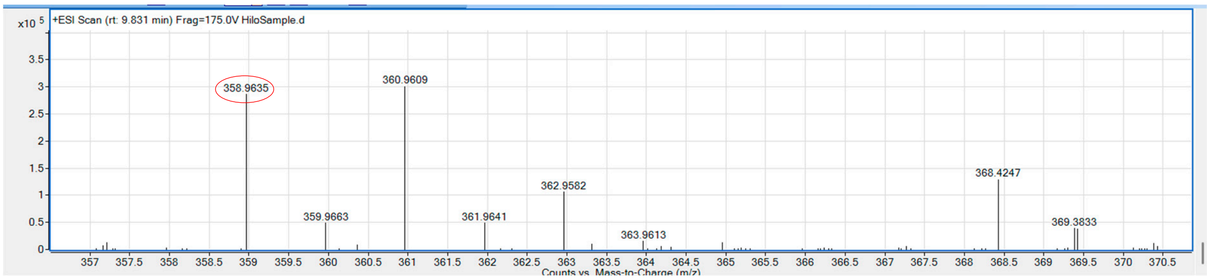

Figure S6. HRESIMS spectrum of clarosione (**1**)

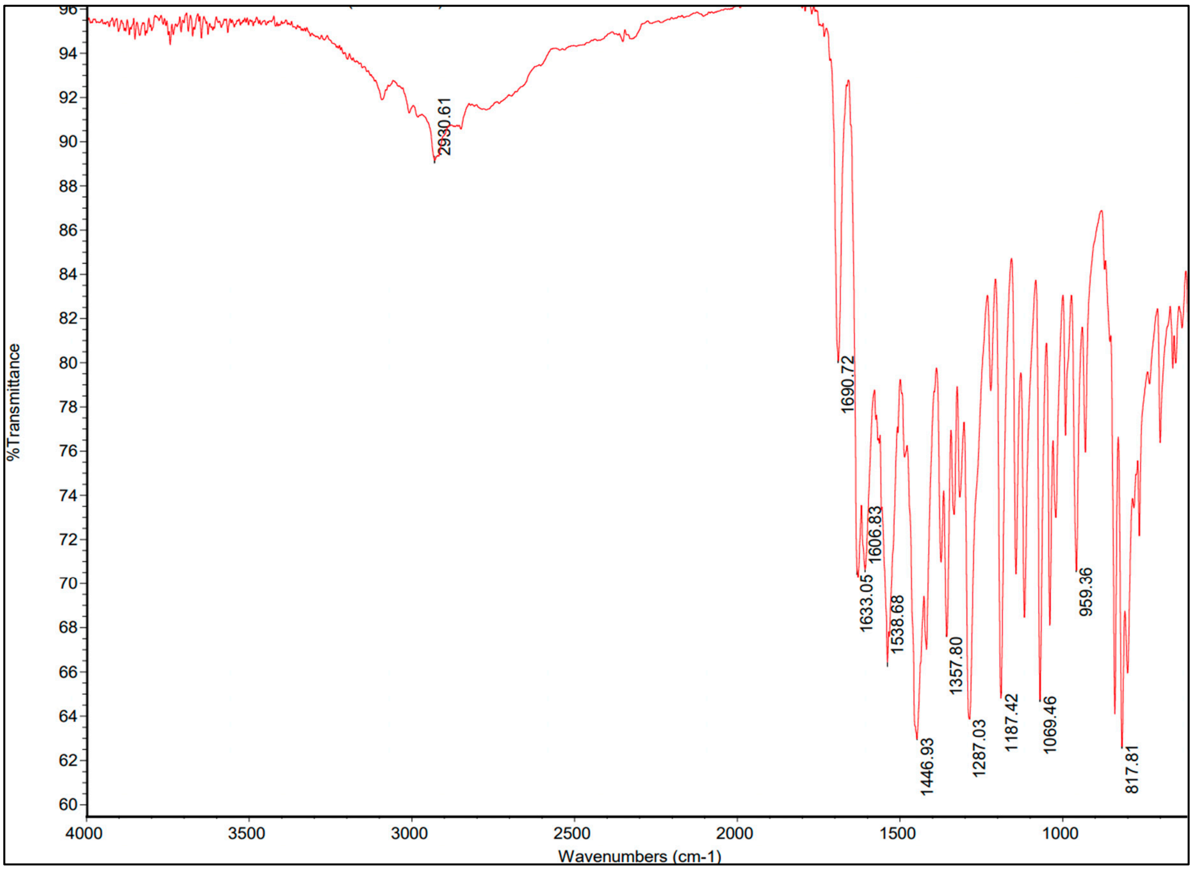

Figure S7. IR radiation spectrum of clarosione (**1**)

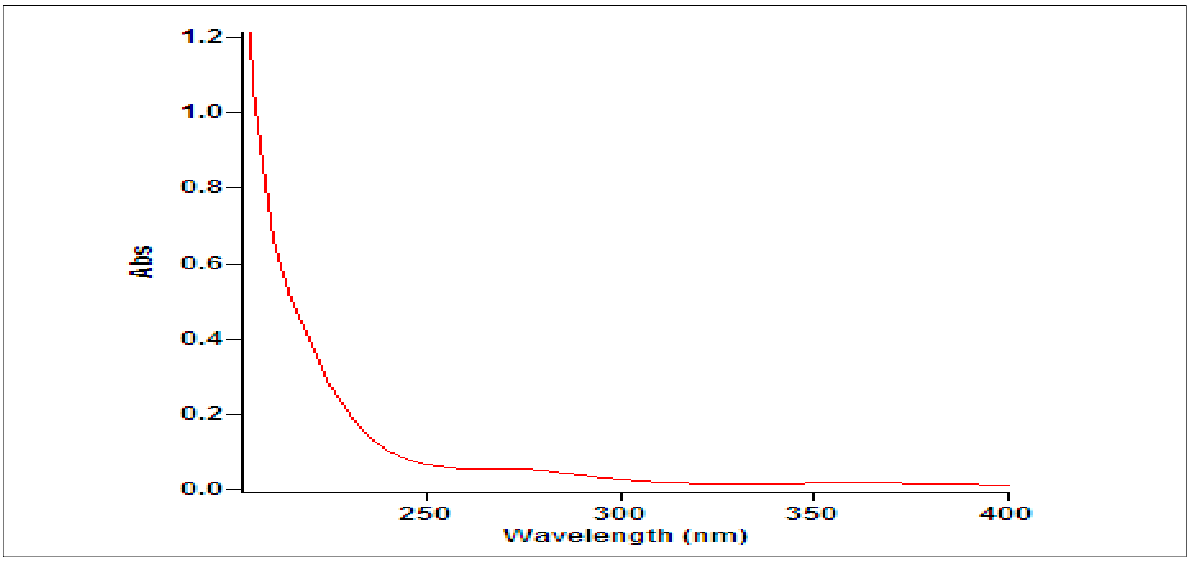

Figure S8. UV spectrum of clarosione (**1**)

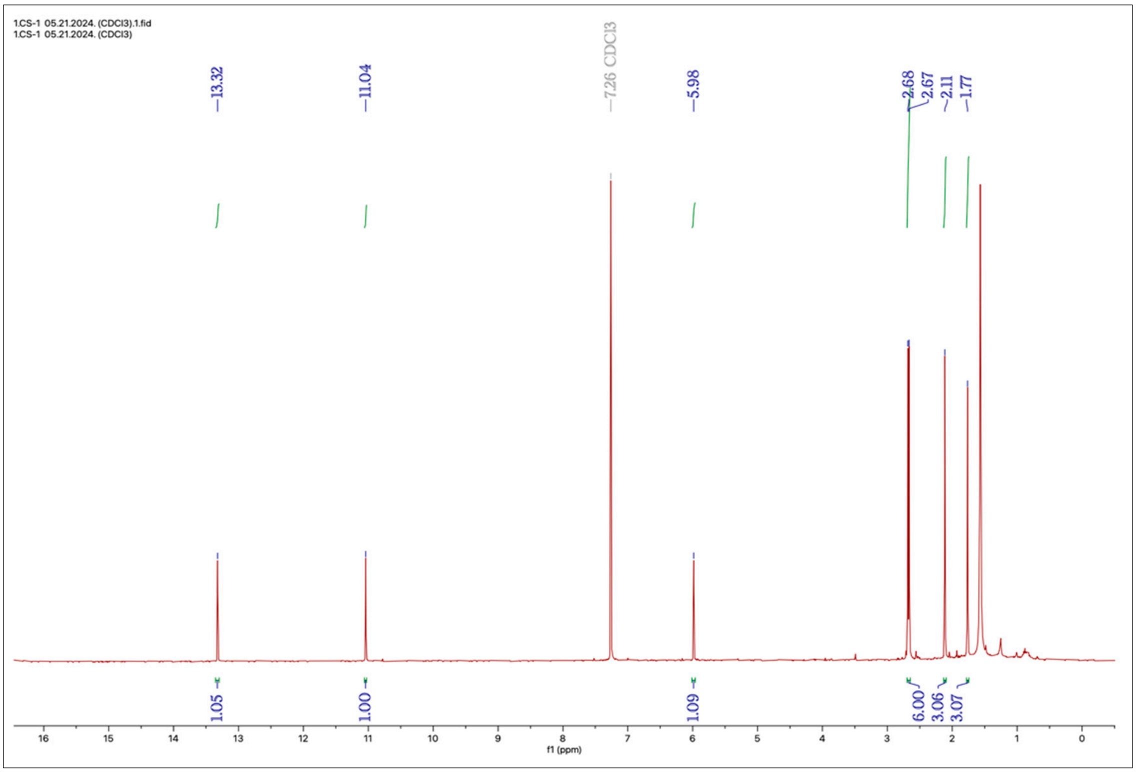

Figure S9. ^{1}H NMR spectrum (400 MHz, $CDCl_3$) of (*S*)-usnic acid (**2**)

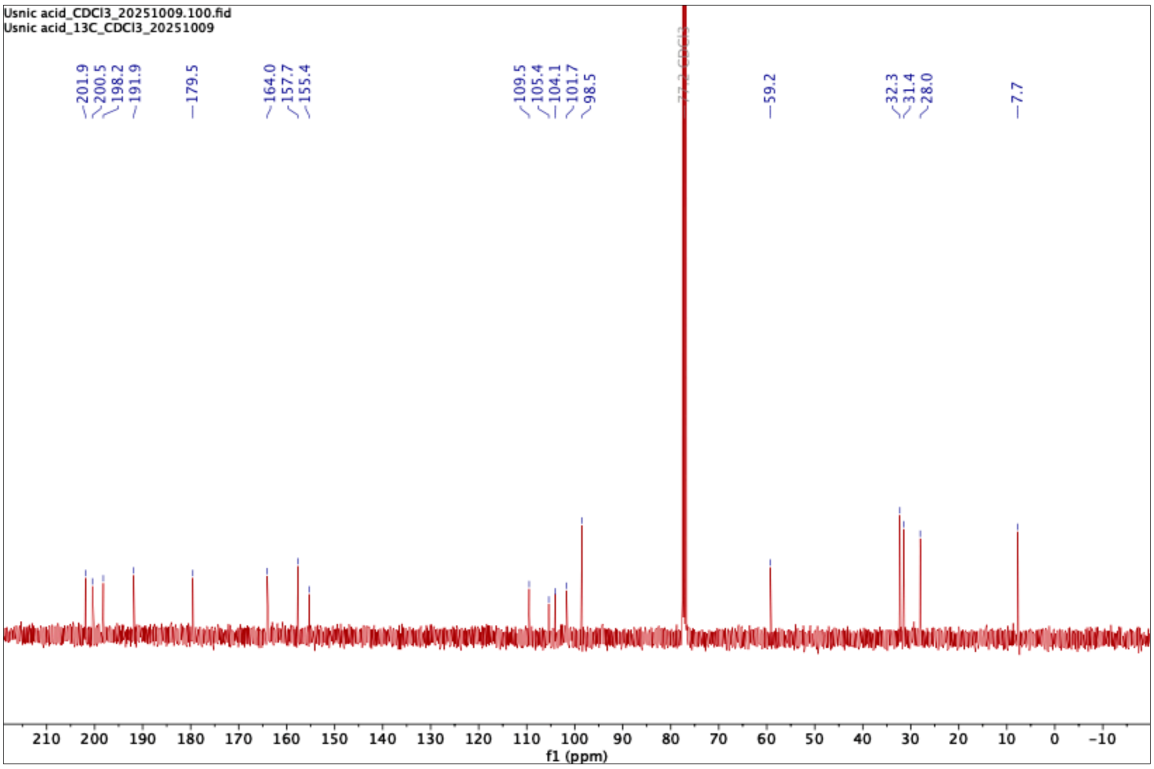

Figure S10. ^{13}C NMR spectrum (100 MHz, $CDCl_3$) of (*S*)-usnic acid (**2**)

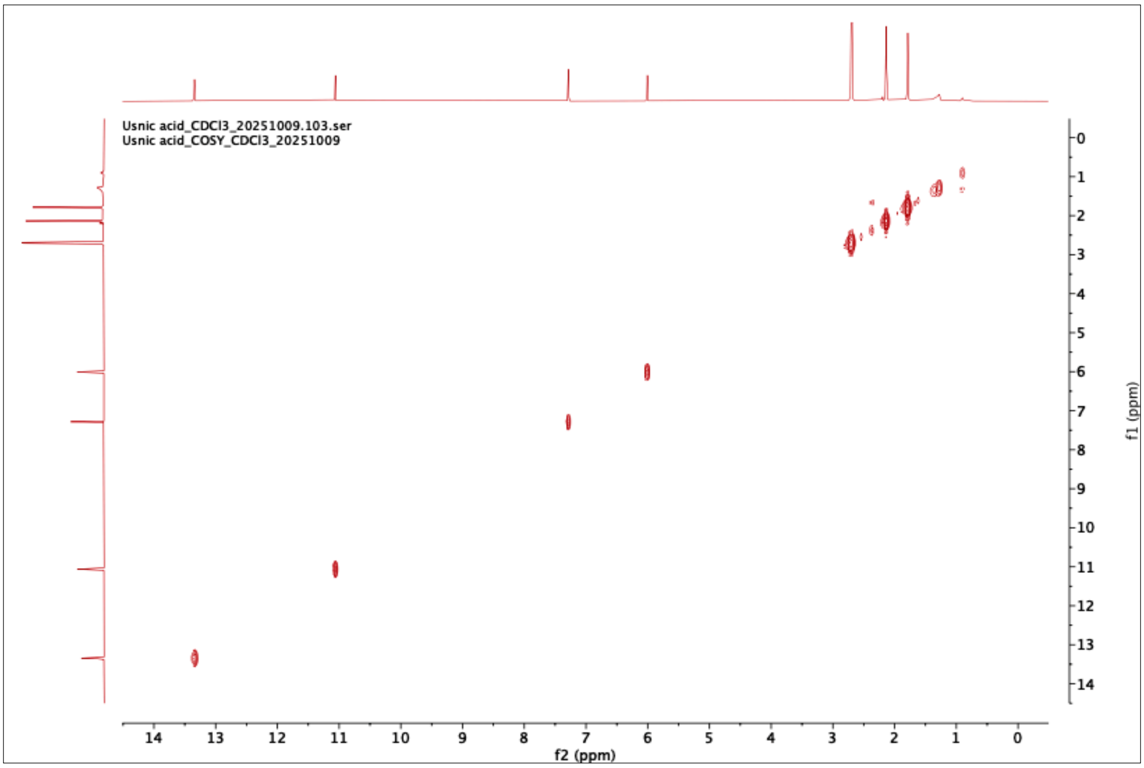

Figure S11. COSY spectrum ($CDCl_3$) of (*S*)-usnic acid (**2**)

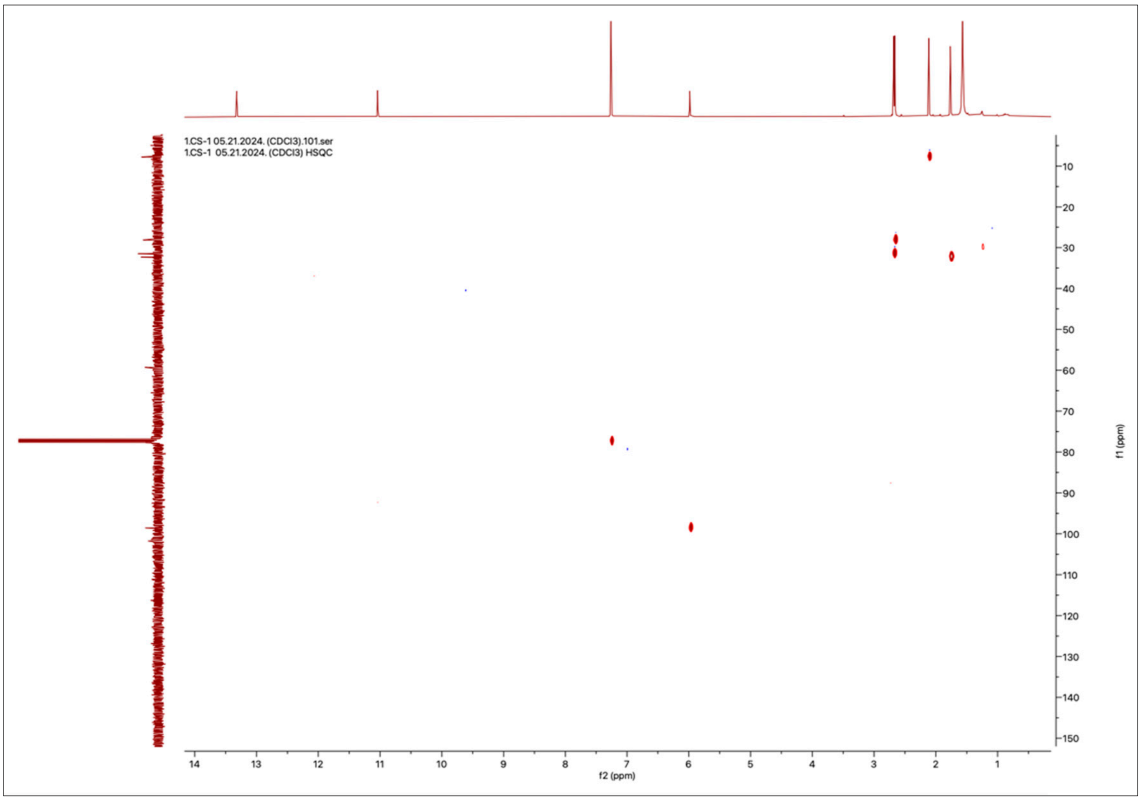

Figure S12. HSQC Spectrum ($CDCl_3$) of (*S*)-usnic acid (**2**)

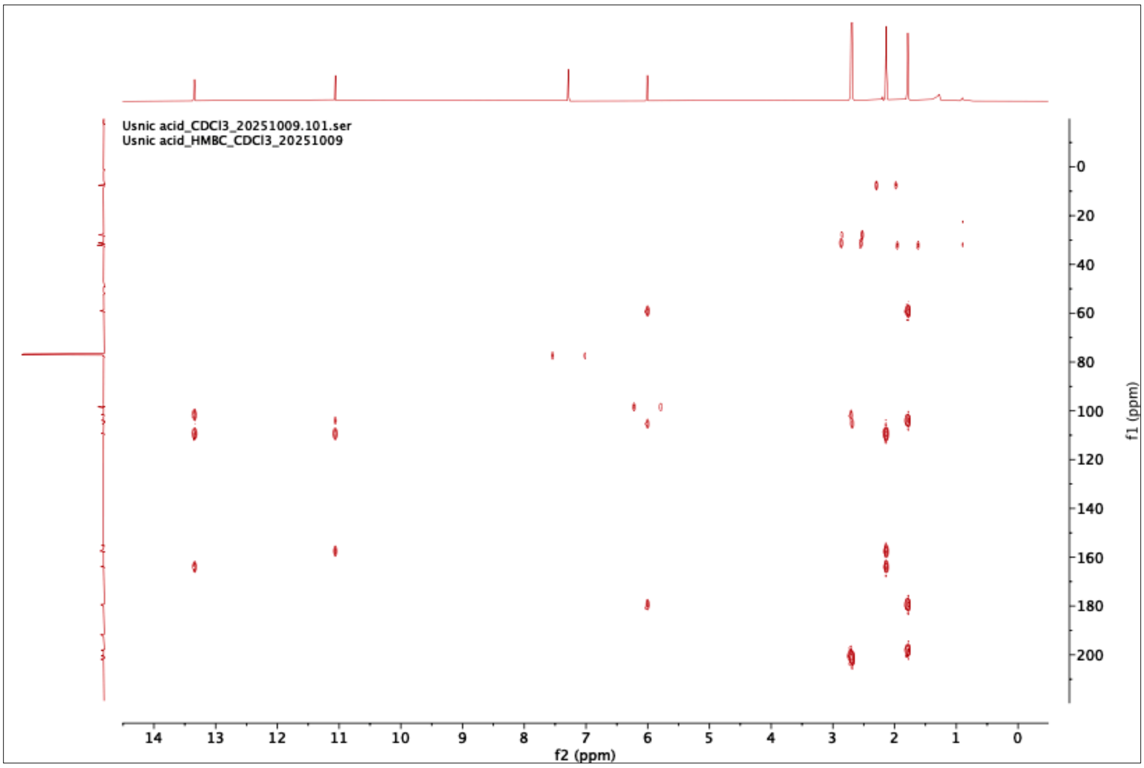

Figure S13. HMBC spectrum ($CDCl_3$) of (*S*)-usnic acid (**2**)

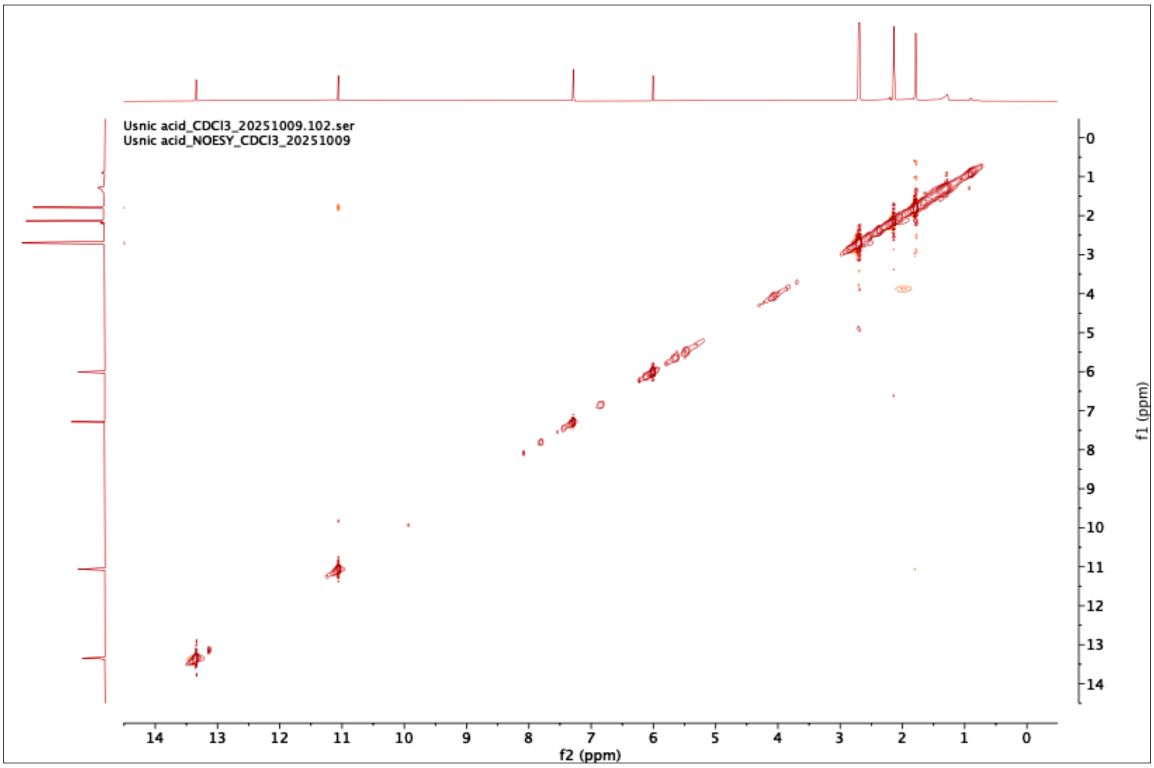

Figure S14. NOESY spectrum ($CDCl_3$) of (*S*)-usnic acid (**2**)

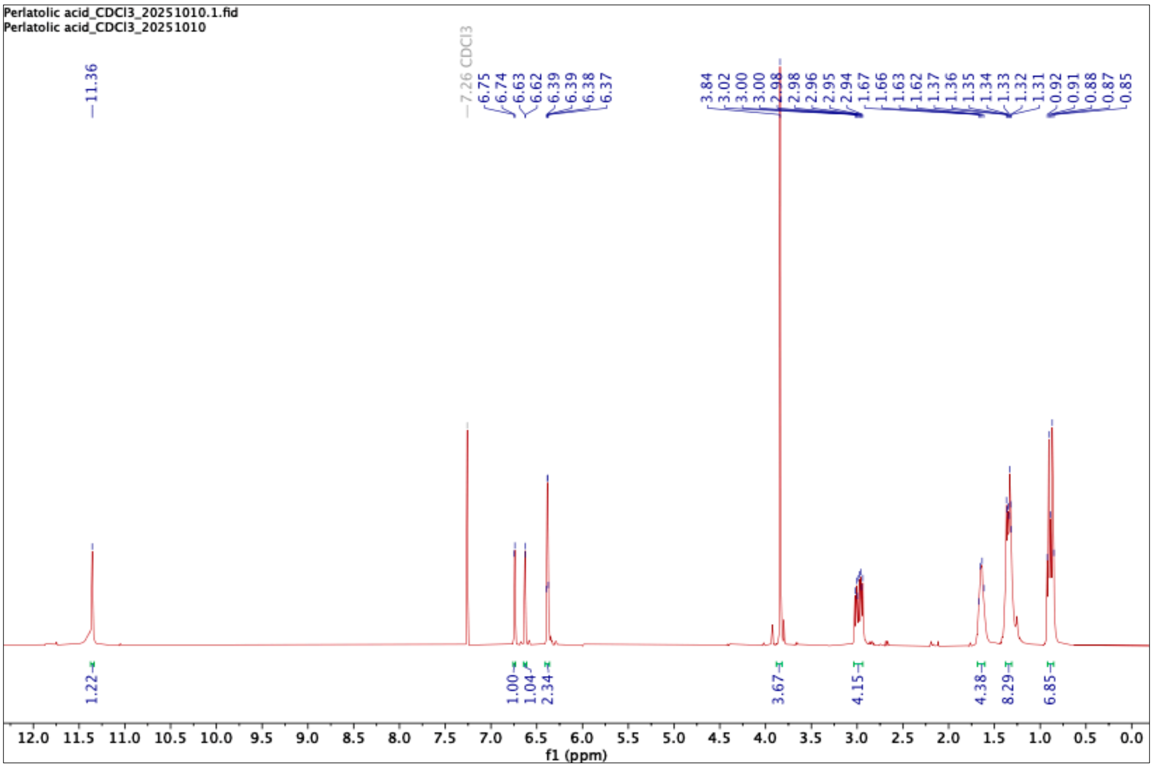

Figure S15. ^{1}H NMR spectrum (400 MHz, $CDCl_3$) of perlatolic acid (**3**)

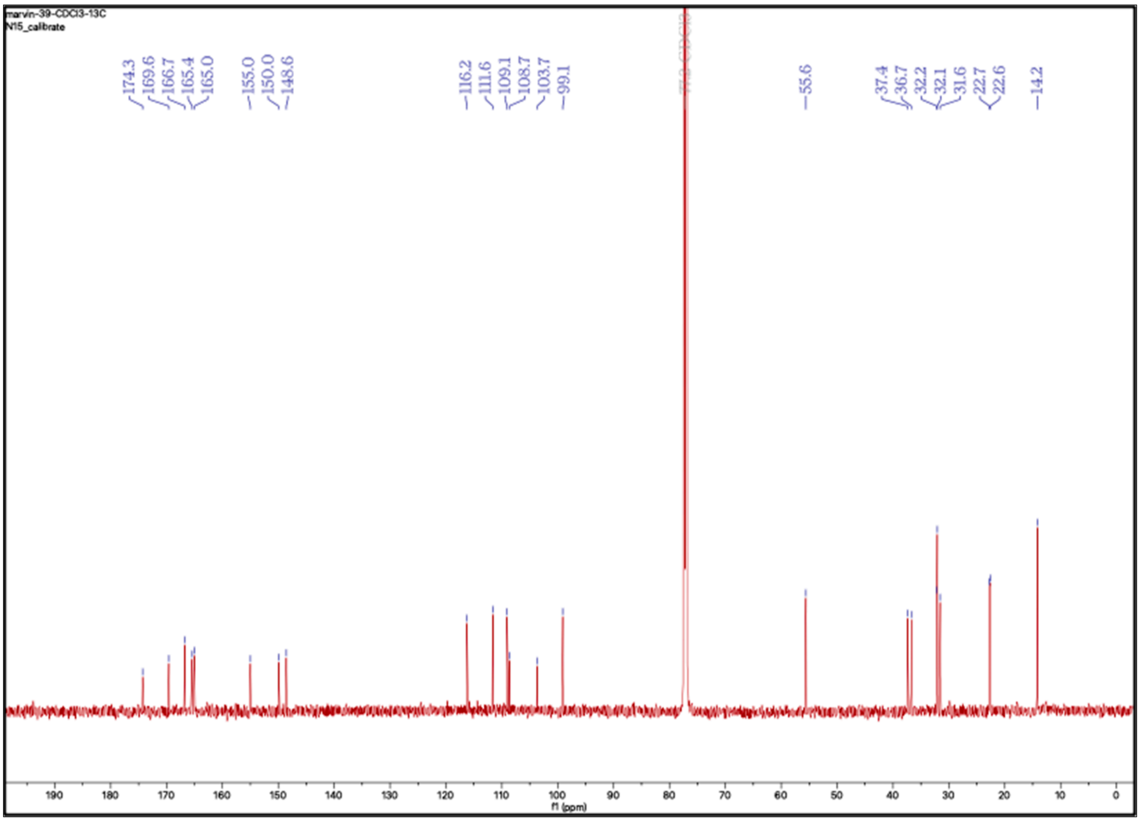

Figure S16. ^{13}C NMR spectrum (100 MHz, $CDCl_3$) of perlatolic acid (**3**)

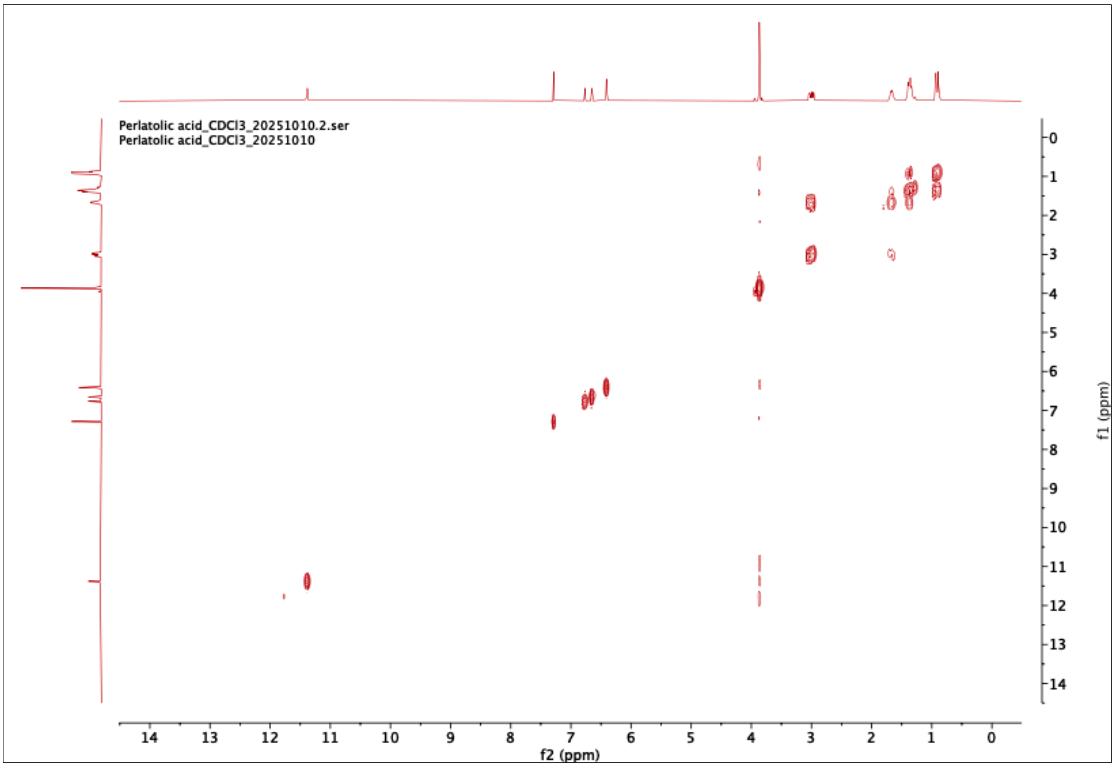

Figure S17. COSY spectrum ($CDCl_3$) of perlatolic acid (**3**)

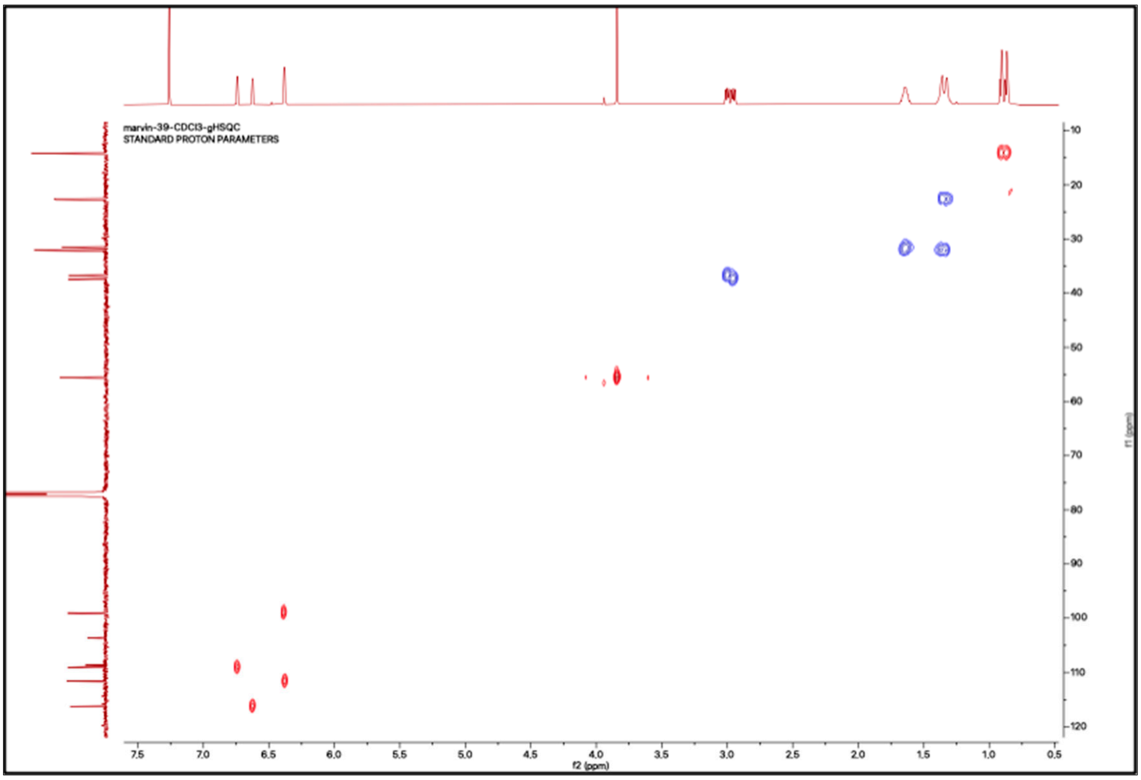

Figure S18. HSQC spectrum ($CDCl_3$) of perlatolic acid (**3**)

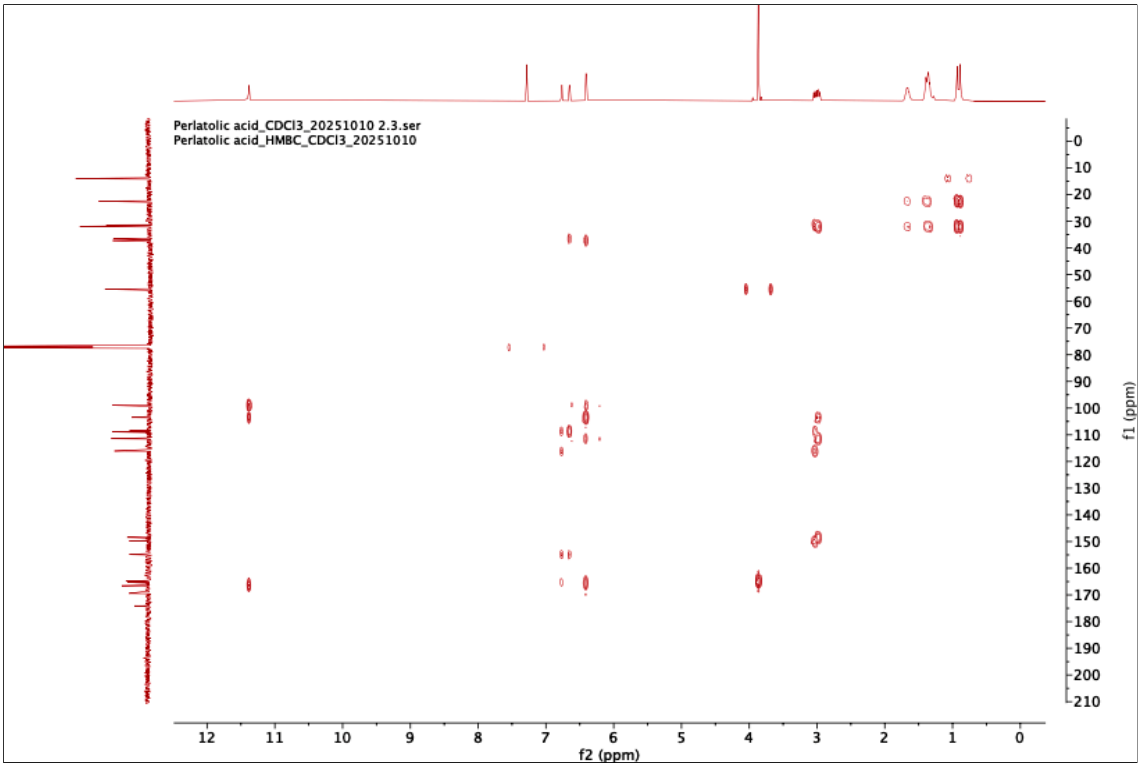

Figure S19. HMBC spectrum ($CDCl_3$) of perlatolic acid (**3**)

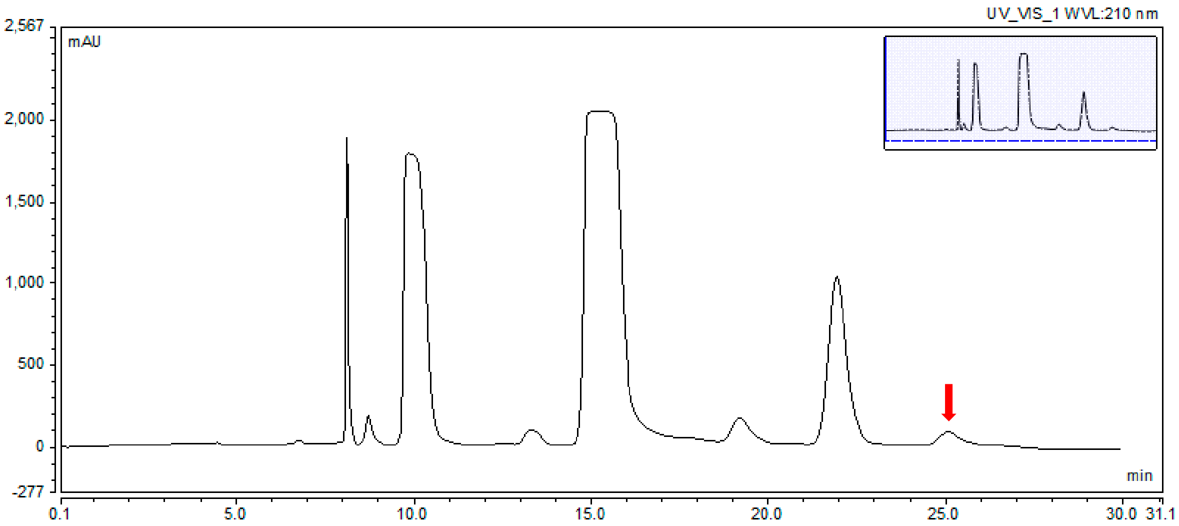

Figure S20. RP–High-performance liquid chromatograms of clarosione (**1**) t_R 25.2, 0.9 mg, MeCN–H_2O, 70:30 to 90:10, flow rate 1.7 mL/min.

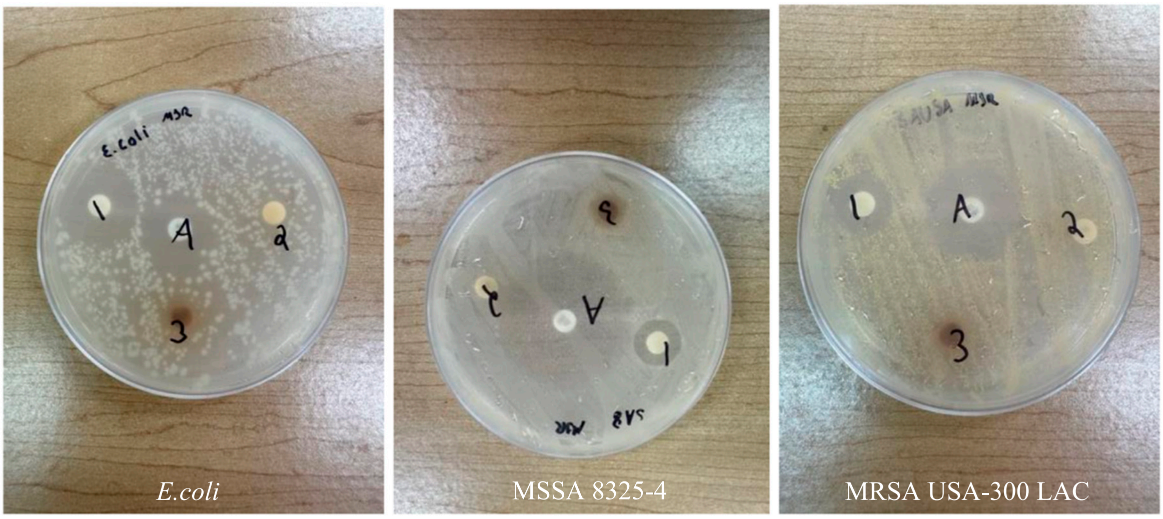

Figure S21. *C. skottsbergii* extract [1] using disk diffusion assay

Crystal Structure Report for Clarosione (1)

A specimen of $C_{15}H_9Cl_3O_4$, approximate dimensions 0.060 mm x 0.060 mm x 0.200 mm, was used for the X-ray crystallographic analysis. The X-ray intensity data were measured on a Bruker D8 VENTURE κ-geometry Bruker D8 VENTURE diffractometer equipped with a Incoatec IμS 3.0 microfocus sealed tube (Cu Kα, λ = 1.54178 Å) and a multilayer mirror monochromator.

Table S1: Data collection details for clarosione (1).

Axis	dx/mm	2θ/°	ω/°	φ/°	χ/°	Width/°	Frames	Time/s
Phi	40.060	55.00	0.00	0.00	54.74	1.00	180	0.50
Phi	45.060	55.00	0.00	0.00	54.74	1.00	180	0.50
Phi	50.060	55.00	0.00	0.00	54.74	1.00	180	0.50
Phi	50.060	55.00	0.00	0.00	54.74	1.00	180	0.50
Phi	52.060	55.00	0.00	0.00	54.74	1.00	180	0.50
Omega	40.060	107.62	-21.95	-160.00	80.00	1.00	89	10.00
Omega	40.060	107.62	-21.95	40.00	80.00	1.00	89	10.00
Omega	40.060	107.62	-21.95	120.00	80.00	1.00	89	10.00
Phi	40.060	48.14	47.65	-173.65	-22.00	1.00	195	3.00
Omega	40.060	107.62	-21.95	160.00	80.00	1.00	89	10.00
Omega	40.060	107.62	-21.95	80.00	80.00	1.00	89	10.00
Omega	40.060	107.62	-21.95	0.00	80.00	1.00	89	10.00
Omega	40.060	107.62	-21.95	-80.00	80.00	1.00	89	10.00
Omega	40.060	107.62	-9.83	-40.00	65.50	1.00	96	10.00
Omega	40.060	107.62	-9.83	160.00	65.50	1.00	96	10.00
Omega	40.060	107.62	-9.83	0.00	65.50	1.00	96	10.00
Phi	40.060	107.62	107.13	-41.38	-22.00	1.00	258	10.00
Omega	40.060	107.62	-9.83	-80.00	65.50	1.00	96	10.00
Omega	40.060	107.62	-9.83	80.00	65.50	1.00	96	10.00
Phi	40.060	48.14	-47.12	-261.65	22.00	1.00	195	3.00
Phi	40.060	48.14	46.22	58.35	-44.50	1.00	195	3.00
Omega	40.060	107.62	-9.83	40.00	65.50	1.00	96	10.00
Omega	40.060	107.62	-9.83	-120.00	65.50	1.00	96	10.00
Phi	40.060	107.62	12.36	-49.38	22.00	1.00	234	10.00
Omega	40.060	107.62	-9.83	-160.00	65.50	1.00	96	10.00
Phi	40.060	47.62	45.70	0.00	-44.50	1.00	360	3.00
Omega	40.060	107.62	-9.83	120.00	65.50	1.00	96	10.00

A total of 3824 frames were collected at 298 K. The total exposure time was 6.41 hours. The frames were integrated with the Bruker SAINT software package using a narrow-frame algorithm. The integration of the data using an orthorhombic unit cell yielded a total of 37383 reflections to a maximum θ angle of 77.27° (0.79 Å resolution), of which 3089 were independent (average redundancy 12.102, completeness = 99.9%, R_{int} = 8.27%, R_{sig} = 3.63%) and 2790 (90.32%) were greater than $2\sigma(F^2)$. The final cell constants of a = 4.37920(10) Å, b = 17.3020(3) Å, c = 19.1464(3) Å, volume = 1450.70(5) $Å^3$, are based upon the refinement of the XYZ-centroids of 8917 reflections above 20 σ(I) with 10.56° < 2θ < 150.1°. Data were corrected for absorption effects using the Multi-Scan method (SADABS). The ratio of minimum to maximum apparent transmission was 0.792. The calculated minimum and maximum transmission coefficients (based on crystal size) are 0.3860 and 0.7200.

The structure was solved and refined using the Bruker SHELXTL Software Package (APEX5), using the space group $P2_12_12_1$, with Z = 4 for the formula unit, $C_{15}H_9Cl_3O_4$. The final anisotropic full-matrix least-squares refinement on F^2 with 207 variables converged at R1 = 3.30%, for the observed data and wR2 = 8.53% for all data. The goodness-of-fit was 1.064. The largest peak in the final difference electron density synthesis was 0.304

e⁻/Å^3 and the largest hole was -0.326 e⁻/Å^3 with an RMS deviation of 0.044 e⁻/Å^3. On the basis of the final model, the calculated density was 1.646 g/cm^3 and F(000), 728 e⁻.

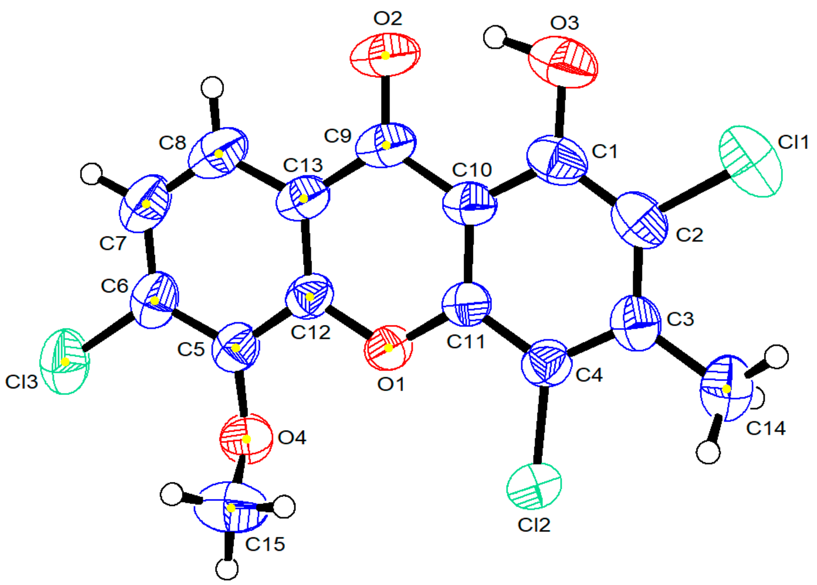

Table S2. Sample and crystal data for clarosione (1).		
**Identification code**	Clarosione	
**Chemical formula**	$C_{15}H_9Cl_3O_4$	
**Formula weight**	359.57 g/mol	
**Temperature**	298(2) K	
**Wavelength**	1.54178 Å	
**Crystal size**	0.060 x 0.060 x 0.200 mm	
**Crystal system**	orthorhombic	
**Space group**	$P2_12_12_1$	
**Unit cell dimensions**	a = 4.37920(10) Å	α = 90°
	b = 17.3020(3) Å	β = 90°
	c = 19.1464(3) Å	γ = 90°
**Volume**	1450.70(5) Å^3	
**Z**	4	
**Density (calculated)**	1.646 g/cm^3	
**Absorption coefficient**	5.870 mm^{-1}	
**F(000)**	728	

Table S3. Data collection and structure refinement for clarosione (1).	
**Diffractometer**	Bruker D8 VENTURE κ-geometry diffractometer
**Radiation source**	Incoatec IμS 3.0 microfocus sealed tube (Cu Kα, λ = 1.54178 Å)
**Theta range for data collection**	3.44 to 77.27°
**Index ranges**	-5<=h<=5, -21<=k<=21, -22<=l<=24
**Reflections collected**	37383
**Independent reflections**	3089 [R(int) = 0.0827]
**Coverage of independent reflections**	99.9%
**Absorption correction**	Multi-Scan
**Max. and min. transmission**	0.5973and 0.7541
**Structure solution technique**	direct methods

**Structure solution program**	XT, VERSION 2019/1 (Sheldrick, 2019)	
**Refinement method**	Full-matrix least-squares on F^2	
**Refinement program**	SHELXTL-2019/1 (Sheldrick, 2019)	
**Function minimized**	Σ w(F_o^2 - F_c^2)^2	
**Data / restraints / parameters**	3089 / 0 / 207	
**Goodness-of-fit on F^2**	1.064	
**Δ/σ_{max}**	0.001	
**Final R indices**	2790 data; I>2σ(I)	R1 = 0.0330, wR2 = 0.0818
	all data	R1 = 0.0378, wR2 = 0.0853
**Weighting scheme**	$w=1/[\sigma^2(F_o^2)+(0.0406P)^2+0.1975P]$<br>where $P=(F_o^2+2F_c^2)/3$	
**Absolute structure parameter**	0.051(9)	
**Largest diff. peak and hole**	0.304 and -0.326 eÅ^{-3}	
**R.M.S. deviation from mean**	0.044 eÅ^{-3}	

Table S4. Atomic coordinates and equivalent isotropic atomic displacement parameters (Å^2) for clarosione (1).

U(eq) is defined as one third of the trace of the orthogonalized U_{ij} tensor.

	x/a	y/b	z/c	U(eq)
Cl1	0.1953(2)	0.66824(7)	0.80415(5)	0.0767(3)
O1	0.3613(4)	0.56360(11)	0.58464(10)	0.0470(5)
C1	0.8639(7)	0.5606(2)	0.73870(16)	0.0549(8)
Cl2	0.5355(2)	0.71989(4)	0.56716(4)	0.0615(2)
O2	0.6372(7)	0.40618(15)	0.73153(14)	0.0755(8)
C2	0.9546(7)	0.6379(2)	0.73805(16)	0.0550(7)
Cl3	0.7383(2)	0.37360(6)	0.44769(5)	0.0740(3)
O3	0.9701(7)	0.51297(18)	0.78793(14)	0.0756(7)
C3	0.8536(7)	0.68984(19)	0.68656(16)	0.0509(7)
O4	0.9593(5)	0.52934(13)	0.48349(12)	0.0583(5)
C4	0.6601(7)	0.66164(17)	0.63439(15)	0.0473(6)
C5	0.0671(7)	0.47226(17)	0.52573(16)	0.0486(6)
C6	0.9708(8)	0.39626(19)	0.51763(18)	0.0563(7)
C7	0.0581(9)	0.33857(19)	0.5646(2)	0.0660(9)
C8	0.2489(10)	0.35652(19)	0.6191(2)	0.0663(9)
C9	0.5590(8)	0.45363(18)	0.68612(16)	0.0549(8)
C10	0.6611(7)	0.53369(18)	0.68681(15)	0.0473(7)
C11	0.5607(7)	0.58502(16)	0.63542(14)	0.0428(6)
C12	0.2672(7)	0.48884(16)	0.58082(14)	0.0453(6)
C13	0.3569(7)	0.43245(17)	0.62853(16)	0.0507(7)
C14	0.9445(10)	0.7737(2)	0.6874(2)	0.0687(9)
C15	0.1516(11)	0.5518(3)	0.4280(2)	0.0863(13)

Table S5. Bond lengths (Å) for clarosione (1).

Cl1-C2	1.729(3)	O1-C11	1.358(4)
O1-C12	1.360(3)	C1-O3	1.335(4)

C1-C2	1.395(5)	C1-C10	1.411(4)
Cl2-C4	1.723(3)	O2-C9	1.244(4)
C2-C3	1.406(5)	Cl3-C6	1.727(4)
O3-H3	1.02(4)	C3-C4	1.398(4)
C3-C14	1.504(5)	O4-C5	1.361(4)
O4-C15	1.410(5)	C4-C11	1.396(4)
C5-C6	1.390(4)	C5-C12	1.401(4)
C6-C7	1.396(5)	C7-C8	1.373(6)
C7-H7	0.930000	C8-C13	1.408(5)
C8-H8	0.930000	C9-C10	1.456(5)
C9-C13	1.461(5)	C10-C11	1.396(4)
C12-C13	1.393(4)	C14-H14A	0.960000
C14-H14B	0.960000	C14-H14C	0.960000
C15-H15A	0.960000	C15-H15B	0.960000
C15-H15C	0.960000		

**Table S6. Bond angles (°) for clarosione (1).**			
C11-O1-C12	119.5(2)	O3-C1-C2	119.9(3)
O3-C1-C10	120.9(3)	C2-C1-C10	119.2(3)
C1-C2-C3	122.0(3)	C1-C2-Cl1	117.3(3)
C3-C2-Cl1	120.7(3)	C1-O3-H3	104.7(19)
C4-C3-C2	117.9(3)	C4-C3-C14	120.3(3)
C2-C3-C14	121.7(3)	C5-O4-C15	116.1(3)
C11-C4-C3	120.7(3)	C11-C4-Cl2	117.9(2)
C3-C4-Cl2	121.4(2)	O4-C5-C6	121.0(3)
O4-C5-C12	121.1(3)	C6-C5-C12	117.9(3)
C5-C6-C7	121.4(3)	C5-C6-Cl3	118.7(3)
C7-C6-Cl3	119.9(3)	C8-C7-C6	119.6(3)
C8-C7-H7	120.200000	C6-C7-H7	120.200000
C7-C8-C13	120.8(3)	C7-C8-H8	119.600000
C13-C8-H8	119.600000	O2-C9-C10	122.5(3)
O2-C9-C13	121.9(3)	C10-C9-C13	115.6(3)
C11-C10-C1	119.0(3)	C11-C10-C9	120.1(3)
C1-C10-C9	120.9(3)	O1-C11-C4	116.7(2)
O1-C11-C10	122.2(3)	C4-C11-C10	121.1(3)
O1-C12-C13	123.1(3)	O1-C12-C5	115.1(2)
C13-C12-C5	121.8(3)	C12-C13-C8	118.4(3)
C12-C13-C9	119.3(3)	C8-C13-C9	122.3(3)
C3-C14-H14A	109.500000	C3-C14-H14B	109.500000
H14A-C14-H14B	109.500000	C3-C14-H14C	109.500000
H14A-C14-H14C	109.500000	H14B-C14-H14C	109.500000
O4-C15-H15A	109.500000	O4-C15-H15B	109.500000
H15A-C15-H15B	109.500000	O4-C15-H15C	109.500000
H15A-C15-H15C	109.500000	H15B-C15-H15C	109.500000

**Table S7. Torsion angles (°) for clarosione (1).**			
O3-C1-C2-C3	178.8(3)	C10-C1-C2-C3	-1.0(5)
O3-C1-C2-Cl1	-1.9(4)	C10-C1-C2-Cl1	178.3(2)
C1-C2-C3-C4	-0.9(5)	Cl1-C2-C3-C4	179.8(2)
C1-C2-C3-C14	178.1(3)	Cl1-C2-C3-C14	-1.2(4)

C2-C3-C4-C11	2.7(4)	C14-C3-C4-C11	-176.4(3)
C2-C3-C4-Cl2	-177.6(2)	C14-C3-C4-Cl2	3.4(4)
C15-O4-C5-C6	-99.8(4)	C15-O4-C5-C12	83.3(4)
O4-C5-C6-C7	-174.1(3)	C12-C5-C6-C7	2.9(5)
O4-C5-C6-Cl3	6.0(4)	C12-C5-C6-Cl3	-177.0(2)
C5-C6-C7-C8	-1.5(5)	Cl3-C6-C7-C8	178.5(3)
C6-C7-C8-C13	0.2(6)	O3-C1-C10-C11	-178.7(3)
C2-C1-C10-C11	1.1(4)	O3-C1-C10-C9	0.8(4)
C2-C1-C10-C9	-179.4(3)	O2-C9-C10-C11	-178.5(3)
C13-C9-C10-C11	1.3(4)	O2-C9-C10-C1	2.0(5)
C13-C9-C10-C1	-178.2(3)	C12-O1-C11-C4	176.9(2)
C12-O1-C11-C10	-3.6(4)	C3-C4-C11-O1	176.8(3)
Cl2-C4-C11-O1	-2.9(4)	C3-C4-C11-C10	-2.6(4)
Cl2-C4-C11-C10	177.6(2)	C1-C10-C11-O1	-178.8(3)
C9-C10-C11-O1	1.7(4)	C1-C10-C11-C4	0.7(4)
C9-C10-C11-C4	-178.8(3)	C11-O1-C12-C13	2.4(4)
C11-O1-C12-C5	-178.6(2)	O4-C5-C12-O1	-5.1(4)
C6-C5-C12-O1	177.9(3)	O4-C5-C12-C13	173.8(3)
C6-C5-C12-C13	-3.2(4)	O1-C12-C13-C8	-179.2(3)
C5-C12-C13-C8	2.0(5)	O1-C12-C13-C9	0.6(4)
C5-C12-C13-C9	-178.2(3)	C7-C8-C13-C12	-0.4(5)
C7-C8-C13-C9	179.8(3)	O2-C9-C13-C12	177.4(3)
C10-C9-C13-C12	-2.4(4)	O2-C9-C13-C8	-2.8(5)
C10-C9-C13-C8	177.4(3)		

Table S8. Anisotropic atomic displacement parameters (Å^2) for clarosione (1).

The anisotropic atomic displacement factor exponent takes the form: $-2\pi^2[h^2 a^{*2} U_{11} + ... + 2 h k a^* b^* U_{12}]$

	U_{11}	U_{22}	U_{33}	U_{23}	U_{13}	U_{12}
Cl1	0.0622(5)	0.1136(8)	0.0543(4)	-0.0134(5)	-0.0046(4)	-0.0055(5)
O1	0.0530(11)	0.0401(10)	0.0479(10)	0.0030(8)	0.0007(9)	0.0013(8)
C1	0.0520(16)	0.070(2)	0.0428(15)	0.0060(14)	0.0045(12)	0.0126(15)
Cl2	0.0818(5)	0.0465(4)	0.0563(4)	0.0100(3)	-0.0032(4)	-0.0025(4)
O2	0.092(2)	0.0631(15)	0.0716(15)	0.0269(13)	-0.0034(14)	0.0159(14)
C2	0.0469(15)	0.073(2)	0.0447(15)	-0.0049(14)	0.0036(13)	0.0043(15)
Cl3	0.0740(6)	0.0695(5)	0.0787(6)	-0.0177(4)	0.0025(5)	-0.0173(5)
O3	0.0779(17)	0.0886(19)	0.0603(14)	0.0140(13)	-0.0144(13)	0.0162(16)
C3	0.0483(15)	0.0563(17)	0.0480(15)	-0.0070(12)	0.0068(13)	-0.0001(13)
O4	0.0584(12)	0.0570(12)	0.0594(12)	0.0015(10)	-0.0041(11)	0.0049(11)
C4	0.0507(15)	0.0456(14)	0.0456(13)	0.0023(12)	0.0063(12)	0.0012(13)
C5	0.0485(15)	0.0452(14)	0.0522(15)	-0.0016(12)	0.0077(13)	0.0019(13)
C6	0.0543(17)	0.0509(17)	0.0637(18)	-0.0077(14)	0.0126(16)	-0.0044(14)
C7	0.076(2)	0.0430(15)	0.079(2)	-0.0027(16)	0.014(2)	-0.0076(16)
C8	0.078(2)	0.0448(17)	0.076(2)	0.0120(15)	0.014(2)	0.0036(16)
C9	0.0604(17)	0.0503(16)	0.0541(16)	0.0137(13)	0.0125(15)	0.0145(14)
C10	0.0488(15)	0.0496(16)	0.0435(14)	0.0071(12)	0.0029(12)	0.0095(13)
C11	0.0456(13)	0.0422(14)	0.0407(13)	0.0023(11)	0.0056(11)	0.0062(12)
C12	0.0507(14)	0.0388(13)	0.0464(14)	0.0015(11)	0.0094(13)	0.0035(12)
C13	0.0559(17)	0.0421(15)	0.0541(16)	0.0081(13)	0.0113(14)	0.0083(13)
C14	0.073(2)	0.062(2)	0.071(2)	-0.0146(17)	0.0017(19)	-0.0070(19)

	U_{11}	U_{22}	U_{33}	U_{23}	U_{13}	U_{12}
C15	0.096(3)	0.093(3)	0.070(2)	0.029(2)	0.008(2)	0.004(2)

Table S9. Hydrogen atomic coordinates and isotropic atomic displacement parameters (Å^2) for clarosione (1).

	x/a	y/b	z/c	U(eq)
H3	0.873(8)	0.460(2)	0.7766(17)	0.050000
H7	-0.0125	0.2883	0.5589	0.079000
H8	0.3075	0.3180	0.6503	0.080000
H14A	1.1211	0.7802	0.7165	0.103000
H14B	0.7791	0.8041	0.7055	0.103000
H14C	0.9912	0.7902	0.6407	0.103000
H15A	0.3222	0.5802	0.4461	0.129000
H15B	0.0395	0.5837	0.3960	0.129000
H15C	0.2241	0.5066	0.4040	0.129000

Table S10. Hydrogen bond distances (Å) and angles (°) for clarosione (1).

	Donor-H	Acceptor-H	Donor-Acceptor	Angle
O3-H3···O2	1.02(4)	1.64(4)	2.589(4)	151.(3)
C15-H15C···O3#1	0.960000	2.620000	3.345(5)	132.900000

#1 -x + 1.5; 1 -y; z – 0.5

Crystal Structure Report for (*S*)-Usnic acid (2)

A greenish-yellow specimen of $C_{18}H_{16}O_7$, approximate dimensions 0.080 mm x 0.080 mm x 0.120 mm, was used for the X-ray crystallographic analysis. The X-ray intensity data were measured on a κ-geometry Buker D8 VENTURE diffractometer equipped with a Incoatec μS 3.0 microfocus sealed tube (λ = 1.54178 Å) and a multilayer mirror monochromator.

Table S11: Data collection details for (*S*)-usnic acid (2)

Axis	dx/mm	2θ/°	ω/°	φ/°	χ/°	Width/°	Frames	Time/s
Phi	45.030	55.00	0.00	0.00	54.74	1.00	180	0.50
Phi	50.030	55.00	0.00	0.00	54.74	1.00	180	0.50
Phi	53.030	55.00	0.00	0.00	54.74	1.00	180	0.50
Phi	40.030	107.62	12.36	-49.38	22.00	1.00	242	12.00
Omega	40.030	107.62	-9.83	40.00	65.50	1.00	96	12.00
Phi	40.030	48.14	46.22	-101.65	-44.50	1.00	203	3.10
Omega	40.030	107.62	-9.83	-160.00	65.50	1.00	96	12.00
Omega	40.030	107.62	-9.83	-40.00	65.50	1.00	96	12.00
Omega	40.030	107.62	-9.83	-120.00	65.50	1.00	96	12.00
Omega	40.030	107.62	-9.83	120.00	65.50	1.00	96	12.00
Phi	40.030	107.62	107.13	-209.38	-22.00	1.00	234	12.00
Omega	40.030	107.62	-21.95	160.00	80.00	1.00	89	12.00
Omega	40.030	107.62	-21.95	40.00	80.00	1.00	89	12.00
Phi	40.030	48.14	47.65	-253.65	-22.00	1.00	187	3.10
Omega	40.030	107.62	-9.83	160.00	65.50	1.00	96	12.00

Axis	dx/mm	2θ/°	ω/°	φ/°	χ/°	Width/°	Frames	Time/s
Omega	40.030	107.62	-9.83	-80.00	65.50	1.00	96	12.00
Omega	40.030	107.62	-9.83	80.00	65.50	1.00	96	12.00
Omega	40.030	107.62	-21.95	120.00	80.00	1.00	89	12.00
Phi	40.030	47.62	45.70	0.00	-44.50	1.00	360	3.00
Omega	40.030	107.62	-9.83	0.00	65.50	1.00	96	12.00
Phi	40.030	48.14	-47.12	18.35	22.00	1.00	187	3.10
Phi	40.030	107.62	105.70	-65.38	-44.50	1.00	266	12.00

A total of 3350 frames were collected at 298 K. The total exposure time was 7.12 hours. The frames were integrated with the Bruker SAINT software package using a narrow-frame algorithm. The integration of the data using an orthorhombic unit cell yielded a total of 76769 reflections to a maximum θ angle of 77.65° (0.79 Å resolution), of which 6638 were independent (average redundancy 11.565, completeness = 99.5%, R_{int} = 5.25%, R_{sig} = 2.15%) and 6273 (94.50%) were greater than 2σ(F^2). The final cell constants of a = 8.06570(10) Å, b = 19.0635(3) Å, c = 20.3423(3) Å, volume = 3127.84(8) $Å^3$, are based upon the refinement of the XYZ-centroids of 9022 reflections above 20 σ(I) with 6.354° < 2θ < 154.8°. Data were corrected for absorption effects using the Multi-Scan method (SADABS); the minimum and maximum transmission coefficients are 0.691 and 0.754.

The structure was solved and refined using the Bruker SHELXTL Software Package (APEX5) using the space group $P2_12_12_1$, with Z = 8 for the formula unit, $C_{18}H_{16}O_7$. The final anisotropic full-matrix least-squares refinement on F^2 with 485 variables converged at R1 = 2.68%, for the observed data and wR2 = 7.69% for all data. The goodness-of-fit was 1.032. The largest peak in the final difference electron density synthesis was 0.167 $e^-/Å^3$ and the largest hole was -0.108 $e^-/Å^3$ with an RMS deviation of 0.027 $e^-/Å^3$. On the basis of the final model, the calculated density was 1.462 g/cm^3 and F(000), 1440 e^-.

The supplementary crystallography data for this paper can be found in the CCDC database under accession number 2389503. These data are available for free at www.ccdc.ac.uk/data_request/cif, or by emailing data_request@ccdc.cam.ac.uk, or by contacting The Cambridge Crystallographic Data Centre, 12 Union Road, Cambridge CB2 1EZ, UK; fax: +44 1223 336033.

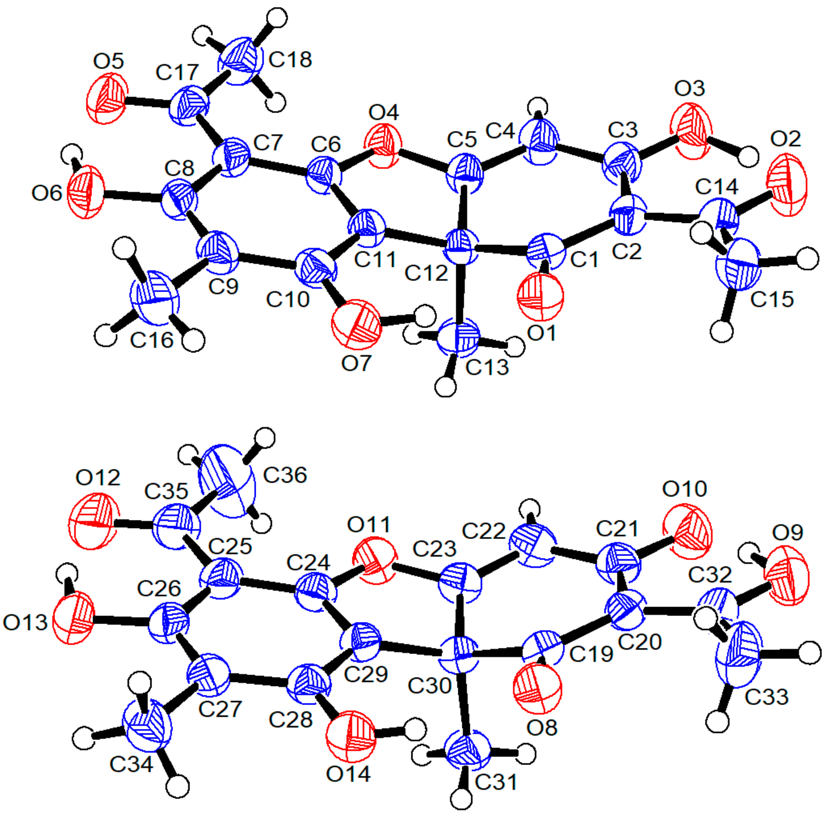

**Table S12. Sample and crystal data for (*S*)-usnic acid (2)**		
**Identification code**	Usnic acid	
**Chemical formula**	$C_{18}H_{16}O_7$	
**Formula weight**	344.31 g/mol	
**Temperature**	298(2) K	
**Wavelength**	1.54178 Å	
**Crystal size**	0.080 x 0.080 x 0.120 mm	
**Crystal system**	orthorhombic	
**Space group**	P 21 21 21	
**Unit cell dimensions**	a = 8.06570(10) Å	α = 90°
	b = 19.0635(3) Å	β = 90°
	c = 20.3423(3) Å	γ = 90°
**Volume**	3127.84(8) $Å^3$	
**Z**	8	
**Density (calculated)**	1.462 g/cm^3	
**Absorption coefficient**	0.961 mm^{-1}	
**F(000)**	1440	

**Table S13. Data collection and structure refinement for *S*)-usnic acid (2).**		
**Theta range for data collection**	3.18 to 77.65°	
**Index ranges**	-10<=h<=10, -23<=k<=24, -24<=l<=25	
**Reflections collected**	76769	
**Independent reflections**	6638 [R(int) = 0.0525]	
**Coverage of independent reflections**	99.5%	
**Absorption correction**	Multi-Scan	
**Max. and min. transmission**	0.9270 and 0.8930	
**Structure solution technique**	direct methods	
**Structure solution program**	XT, VERSION 2018/2	
**Refinement method**	Full-matrix least-squares on F^2	
**Refinement program**	SHELXL-2019/1 (Sheldrick, 2019)	
**Function minimized**	$\Sigma w(F_o^2 - F_c^2)^2$	
**Data / restraints / parameters**	6638 / 0 / 485	
**Goodness-of-fit on F^2**	1.032	
**Δ/σ_{max}**	0.093	
**Final R indices**	6273 data; I>2σ(I)	R1 = 0.0268, wR2 = 0.0751
	all data	R1 = 0.0288, wR2 = 0.0769
**Weighting scheme**	$w=1/[\sigma^2(F_o^2)+(0.0503P)^2+0.1406P]$ where $P=(F_o^2+2F_c^2)/3$	
**Absolute structure parameter**	0.02(4)	
**Extinction coefficient**	0.0010(2)	
**Largest diff. peak and hole**	0.167 and -0.108 $eÅ^{-3}$	
**R.M.S. deviation from mean**	0.027 $eÅ^{-3}$	

**Table S14. Atomic coordinates and equivalent isotropic atomic displacement parameters ($Å^2$) for *S*)-usnic acid (2).**
U(eq) is defined as one third of the trace of the orthogonalized U_{ij} tensor.

	x/a	y/b	z/c	U(eq)
O1	0.32168(17)	0.81024(6)	0.19333(6)	0.0452(4)
C1	0.35053(19)	0.75635(8)	0.16128(7)	0.0332(4)
O2	0.0474(2)	0.74943(9)	0.02765(8)	0.0681(4)
C2	0.2616(2)	0.73435(8)	0.10394(7)	0.0363(4)
O3	0.2219(2)	0.64719(8)	0.02270(7)	0.0612(4)
C3	0.3016(2)	0.66817(9)	0.07374(8)	0.0412(5)
O4	0.58969(15)	0.59752(6)	0.19537(5)	0.0406(4)
C4	0.4189(3)	0.61983(9)	0.10176(8)	0.0447(5)
O5	0.84403(18)	0.51771(8)	0.36257(7)	0.0584(4)
C5	0.5014(2)	0.64053(8)	0.15523(7)	0.0352(5)
O6	0.79192(18)	0.63512(9)	0.41723(6)	0.0543(5)
C6	0.60998(19)	0.63379(8)	0.25493(7)	0.0332(5)
O7	0.49555(19)	0.81022(7)	0.30466(7)	0.0510(5)
C7	0.6929(2)	0.60601(9)	0.30954(7)	0.0379(4)
O8	0.42878(18)	0.63558(7)	0.48636(6)	0.0478(4)
C8	0.7068(2)	0.65393(9)	0.36312(7)	0.0394(5)
O9	0.7757(2)	0.49860(10)	0.57238(8)	0.0681(4)
C9	0.6371(2)	0.72120(10)	0.36302(8)	0.0423(5)
O10	0.6813(2)	0.41169(8)	0.49454(8)	0.0636(4)
C10	0.5573(2)	0.74483(9)	0.30596(7)	0.0373(4)
O11	0.31104(17)	0.47465(6)	0.32591(6)	0.0473(4)
C11	0.54587(19)	0.70043(8)	0.25137(7)	0.0335(4)
O12	0.0771(2)	0.53040(11)	0.14550(7)	0.0756(6)
C12	0.50375(19)	0.71430(8)	0.17997(7)	0.0318(5)
O13	0.01253(19)	0.64790(9)	0.19620(7)	0.0585(5)
C13	0.6532(2)	0.75313(10)	0.14697(8)	0.0441(5)
O14	0.20569(19)	0.69958(7)	0.40873(7)	0.0487(5)
C14	0.1215(2)	0.77239(10)	0.07832(8)	0.0435(5)
C15	0.0521(3)	0.83677(11)	0.10854(11)	0.0524(6)
C16	0.6531(3)	0.76825(12)	0.42207(9)	0.0602(7)
C17	0.7641(2)	0.53602(10)	0.31314(8)	0.0429(5)
C18	0.7424(3)	0.48373(10)	0.25900(11)	0.0562(6)
C19	0.4445(2)	0.57370(8)	0.47007(7)	0.0354(4)
C20	0.5636(2)	0.52549(9)	0.49777(7)	0.0385(4)
C21	0.5794(2)	0.45630(9)	0.47026(9)	0.0429(5)
C22	0.4951(2)	0.43632(9)	0.41040(9)	0.0455(5)
C23	0.3810(2)	0.47971(8)	0.38719(8)	0.0387(5)
C24	0.2429(2)	0.54058(9)	0.31181(8)	0.0378(5)
C25	0.1659(2)	0.55750(10)	0.25217(8)	0.0442(5)
C26	0.0969(2)	0.62639(10)	0.25034(8)	0.0426(5)
C27	0.1100(2)	0.67427(9)	0.30200(8)	0.0406(5)
C28	0.1923(2)	0.65327(8)	0.35947(8)	0.0374(4)
C29	0.2569(2)	0.58536(8)	0.36440(7)	0.0359(4)
C30	0.3182(2)	0.54306(8)	0.42242(7)	0.0345(5)
C31	0.1685(2)	0.52204(10)	0.46698(9)	0.0465(6)
C32	0.6743(3)	0.54393(11)	0.54990(8)	0.0477(5)
C33	0.6802(4)	0.61430(12)	0.58035(11)	0.0654(7)
C34	0.0369(3)	0.74636(11)	0.29754(10)	0.0534(6)
C35	0.1524(3)	0.51112(14)	0.19507(10)	0.0585(6)
C36	0.2283(5)	0.43991(19)	0.19482(17)	0.1092(14)

Table S15. Bond lengths (Å) for *S*)-usnic acid (2)

O1-C1	1.2388(19)	C1-C2	1.432(2)
C1-C12	1.521(2)	O2-C14	1.269(2)
O2-H3	1.26(3)	C2-C3	1.440(2)
C2-C14	1.440(2)	O3-C3	1.285(2)
O3-H3	1.19(3)	C3-C4	1.438(3)
O4-C5	1.3586(19)	O4-C6	1.4045(18)
C4-C5	1.335(2)	C4-H4	0.930000
O5-C17	1.244(2)	C5-C12	1.494(2)
O6-C8	1.3462(19)	O6-H6	1.03(3)
C6-C11	1.374(2)	C6-C7	1.401(2)
O7-C10	1.343(2)	O7-H7	0.97(3)
C7-C8	1.426(2)	C7-C17	1.454(2)
O8-C19	1.232(2)	C8-C9	1.400(3)
O9-C32	1.275(2)	O9-H10	1.21(3)
C9-C10	1.402(2)	C9-C16	1.505(2)
O10-C21	1.282(2)	O10-H10	1.25(3)
C10-C11	1.399(2)	O11-C23	1.372(2)
O11-C24	1.401(2)	C11-C12	1.5149(19)
O12-C35	1.233(3)	C12-C13	1.566(2)
O13-C26	1.358(2)	O13-H13	1.09(3)
C13-H13A	0.960000	C13-H13B	0.960000
C13-H13C	0.960000	O14-C28	1.340(2)
O14-H14	0.88(3)	C14-C15	1.483(3)
C15-H15A	0.960000	C15-H15B	0.960000
C15-H15C	0.960000	C16-H16A	0.960000
C16-H16B	0.960000	C16-H16C	0.960000
C17-C18	1.496(3)	C18-H18A	0.960000
C18-H18B	0.960000	C18-H18C	0.960000
C19-C20	1.444(2)	C19-C30	1.523(2)
C20-C32	1.430(2)	C20-C21	1.438(2)
C21-C22	1.446(3)	C22-C23	1.325(3)
C22-H22	0.930000	C23-C30	1.493(2)
C24-C29	1.373(2)	C24-C25	1.401(2)
C25-C26	1.427(3)	C25-C35	1.464(3)
C26-C27	1.396(3)	C27-C28	1.403(2)
C27-C34	1.498(3)	C28-C29	1.399(2)
C29-C30	1.513(2)	C30-C31	1.562(2)
C31-H31A	0.960000	C31-H31B	0.960000
C31-H31C	0.960000	C32-C33	1.478(3)
C33-H33A	0.960000	C33-H33B	0.960000
C33-H33C	0.960000	C34-H34A	0.960000
C34-H34B	0.960000	C34-H34C	0.960000
C35-C36	1.489(4)	C36-H36A	0.960000
C36-H36B	0.960000	C36-H36C	0.960000

**Table S16. Bond angles (°) for (*S*)-usnic acid (2)**			
O1-C1-C2	125.27(15)	O1-C1-C12	117.26(13)
C2-C1-C12	117.13(13)	C14-O2-H3	101.5(14)
C1-C2-C3	119.47(14)	C1-C2-C14	122.70(15)
C3-C2-C14	117.55(15)	C3-O3-H3	100.2(14)
O3-C3-C2	120.35(16)	O3-C3-C4	116.73(15)
C2-C3-C4	122.66(14)	C5-O4-C6	106.41(12)
C5-C4-C3	117.48(15)	C5-C4-H4	121.300000

C3-C4-H4	121.300000	C4-C5-O4	124.94(15)
C4-C5-C12	123.99(15)	O4-C5-C12	111.04(12)
C8-O6-H6	106.7(15)	C11-C6-C7	124.84(14)
C11-C6-O4	111.47(13)	C7-C6-O4	123.62(14)
C10-O7-H7	103.7(17)	C6-C7-C8	113.65(14)
C6-C7-C17	125.12(15)	C8-C7-C17	121.21(14)
O6-C8-C9	116.74(15)	O6-C8-C7	119.61(16)
C9-C8-C7	123.64(14)	C32-O9-H10	101.7(15)
C8-C9-C10	118.68(14)	C8-C9-C16	120.69(16)
C10-C9-C16	120.59(17)	C21-O10-H10	99.8(14)
O7-C10-C11	121.44(14)	O7-C10-C9	119.01(14)
C11-C10-C9	119.54(15)	C23-O11-C24	106.51(12)
C6-C11-C10	119.53(14)	C6-C11-C12	107.24(13)
C10-C11-C12	132.07(14)	C5-C12-C11	99.30(11)
C5-C12-C1	113.69(13)	C11-C12-C1	120.91(12)
C5-C12-C13	108.08(13)	C11-C12-C13	108.72(12)
C1-C12-C13	105.61(12)	C26-O13-H13	99.6(17)
C12-C13-H13A	109.500000	C12-C13-H13B	109.500000
H13A-C13-H13B	109.500000	C12-C13-H13C	109.500000
H13A-C13-H13C	109.500000	H13B-C13-H13C	109.500000
C28-O14-H14	111.2(15)	O2-C14-C2	119.30(17)
O2-C14-C15	116.40(17)	C2-C14-C15	124.27(16)
C14-C15-H15A	109.500000	C14-C15-H15B	109.500000
H15A-C15-H15B	109.500000	C14-C15-H15C	109.500000
H15A-C15-H15C	109.500000	H15B-C15-H15C	109.500000
C9-C16-H16A	109.500000	C9-C16-H16B	109.500000
H16A-C16-H16B	109.500000	C9-C16-H16C	109.500000
H16A-C16-H16C	109.500000	H16B-C16-H16C	109.500000
O5-C17-C7	120.17(17)	O5-C17-C18	117.95(17)
C7-C17-C18	121.88(15)	C17-C18-H18A	109.500000
C17-C18-H18B	109.500000	H18A-C18-H18B	109.500000
C17-C18-H18C	109.500000	H18A-C18-H18C	109.500000
H18B-C18-H18C	109.500000	O8-C19-C20	124.97(15)
O8-C19-C30	118.00(15)	C20-C19-C30	116.71(14)
C32-C20-C21	117.31(16)	C32-C20-C19	123.24(15)
C21-C20-C19	119.38(15)	O10-C21-C20	121.02(17)
O10-C21-C22	116.82(17)	C20-C21-C22	121.85(15)
C23-C22-C21	117.54(16)	C23-C22-H22	121.200000
C21-C22-H22	121.200000	C22-C23-O11	124.45(15)
C22-C23-C30	124.73(16)	O11-C23-C30	110.73(14)
C29-C24-O11	111.47(14)	C29-C24-C25	124.61(16)
O11-C24-C25	123.90(14)	C24-C25-C26	114.05(15)
C24-C25-C35	125.55(18)	C26-C25-C35	120.40(17)
O13-C26-C27	116.83(17)	O13-C26-C25	119.64(17)
C27-C26-C25	123.53(15)	C26-C27-C28	118.48(16)
C26-C27-C34	121.61(16)	C28-C27-C34	119.91(16)
O14-C28-C29	121.75(14)	O14-C28-C27	118.25(15)
C29-C28-C27	119.99(15)	C24-C29-C28	119.28(15)
C24-C29-C30	107.65(14)	C28-C29-C30	132.14(14)
C23-C30-C29	99.65(12)	C23-C30-C19	112.91(14)
C29-C30-C19	120.74(13)	C23-C30-C31	109.45(13)
C29-C30-C31	109.65(13)	C19-C30-C31	104.26(13)
C30-C31-H31A	109.500000	C30-C31-H31B	109.500000
H31A-C31-H31B	109.500000	C30-C31-H31C	109.500000

H31A-C31-H31C	109.500000	H31B-C31-H31C	109.500000
O9-C32-C20	119.99(18)	O9-C32-C33	116.39(18)
C20-C32-C33	123.62(17)	C32-C33-H33A	109.500000
C32-C33-H33B	109.500000	H33A-C33-H33B	109.500000
C32-C33-H33C	109.500000	H33A-C33-H33C	109.500000
H33B-C33-H33C	109.500000	C27-C34-H34A	109.500000
C27-C34-H34B	109.500000	H34A-C34-H34B	109.500000
C27-C34-H34C	109.500000	H34A-C34-H34C	109.500000
H34B-C34-H34C	109.500000	O12-C35-C25	120.4(2)
O12-C35-C36	118.1(2)	C25-C35-C36	121.50(19)
C35-C36-H36A	109.500000	C35-C36-H36B	109.500000
H36A-C36-H36B	109.500000	C35-C36-H36C	109.500000
H36A-C36-H36C	109.500000	H36B-C36-H36C	109.500000

**Table S17. Torsion angles (°) for (*S*)-usnic acid (2).**			
O1-C1-C2-C3	-176.15(16)	C12-C1-C2-C3	10.7(2)
O1-C1-C2-C14	-2.4(3)	C12-C1-C2-C14	-175.60(14)
C1-C2-C3-O3	179.23(16)	C14-C2-C3-O3	5.2(2)
C1-C2-C3-C4	5.2(3)	C14-C2-C3-C4	-168.86(17)
O3-C3-C4-C5	-179.30(18)	C2-C3-C4-C5	-5.1(3)
C3-C4-C5-O4	165.81(16)	C3-C4-C5-C12	-12.0(3)
C6-O4-C5-C4	-161.26(17)	C6-O4-C5-C12	16.83(17)
C5-O4-C6-C11	-4.06(17)	C5-O4-C6-C7	178.95(15)
C11-C6-C7-C8	-0.2(2)	O4-C6-C7-C8	176.43(14)
C11-C6-C7-C17	-178.65(15)	O4-C6-C7-C17	-2.1(2)
C6-C7-C8-O6	-176.30(14)	C17-C7-C8-O6	2.3(2)
C6-C7-C8-C9	3.2(2)	C17-C7-C8-C9	-178.29(15)
O6-C8-C9-C10	175.62(15)	C7-C8-C9-C10	-3.9(2)
O6-C8-C9-C16	-2.1(2)	C7-C8-C9-C16	178.44(17)
C8-C9-C10-O7	-177.55(15)	C16-C9-C10-O7	0.2(3)
C8-C9-C10-C11	1.4(2)	C16-C9-C10-C11	179.14(17)
C7-C6-C11-C10	-2.0(2)	O4-C6-C11-C10	-178.99(14)
C7-C6-C11-C12	167.11(14)	O4-C6-C11-C12	-9.84(17)
O7-C10-C11-C6	-179.68(15)	C9-C10-C11-C6	1.4(2)
O7-C10-C11-C12	14.3(3)	C9-C10-C11-C12	-164.63(16)
C4-C5-C12-C11	156.64(17)	O4-C5-C12-C11	-21.47(16)
C4-C5-C12-C1	26.8(2)	O4-C5-C12-C1	-151.28(12)
C4-C5-C12-C13	-90.1(2)	O4-C5-C12-C13	91.83(15)
C6-C11-C12-C5	18.04(16)	C10-C11-C12-C5	-174.70(17)
C6-C11-C12-C1	142.96(14)	C10-C11-C12-C1	-49.8(2)
C6-C11-C12-C13	-94.76(15)	C10-C11-C12-C13	72.5(2)
O1-C1-C12-C5	161.44(14)	C2-C1-C12-C5	-24.83(18)
O1-C1-C12-C11	43.5(2)	C2-C1-C12-C11	-142.76(14)
O1-C1-C12-C13	-80.24(16)	C2-C1-C12-C13	93.48(15)
C1-C2-C14-O2	-179.27(17)	C3-C2-C14-O2	-5.4(3)
C1-C2-C14-C15	-1.3(3)	C3-C2-C14-C15	172.55(17)
C6-C7-C17-O5	175.75(16)	C8-C7-C17-O5	-2.6(2)
C6-C7-C17-C18	-5.2(3)	C8-C7-C17-C18	176.43(16)
O8-C19-C20-C32	2.5(3)	C30-C19-C20-C32	-170.85(15)
O8-C19-C20-C21	-174.43(16)	C30-C19-C20-C21	12.2(2)
C32-C20-C21-O10	4.4(3)	C19-C20-C21-O10	-178.49(17)
C32-C20-C21-C22	-168.96(17)	C19-C20-C21-C22	8.1(3)
O10-C21-C22-C23	175.40(18)	C20-C21-C22-C23	-11.0(3)

C21-C22-C23-O11	168.24(16)	C21-C22-C23-C30	-7.9(3)
C24-O11-C23-C22	-160.39(18)	C24-O11-C23-C30	16.25(18)
C23-O11-C24-C29	-4.58(18)	C23-O11-C24-C25	177.22(16)
C29-C24-C25-C26	-1.6(2)	O11-C24-C25-C26	176.39(15)
C29-C24-C25-C35	178.77(18)	O11-C24-C25-C35	-3.3(3)
C24-C25-C26-O13	-177.11(15)	C35-C25-C26-O13	2.6(2)
C24-C25-C26-C27	2.6(2)	C35-C25-C26-C27	-177.71(18)
O13-C26-C27-C28	178.26(15)	C25-C26-C27-C28	-1.5(2)
O13-C26-C27-C34	-0.9(2)	C25-C26-C27-C34	179.34(16)
C26-C27-C28-O14	179.51(15)	C34-C27-C28-O14	-1.3(2)
C26-C27-C28-C29	-0.9(2)	C34-C27-C28-C29	178.33(16)
O11-C24-C29-C28	-178.77(14)	C25-C24-C29-C28	-0.6(2)
O11-C24-C29-C30	-8.51(18)	C25-C24-C29-C30	169.66(15)
O14-C28-C29-C24	-178.53(15)	C27-C28-C29-C24	1.9(2)
O14-C28-C29-C30	14.0(3)	C27-C28-C29-C30	-165.55(16)
C22-C23-C30-C29	156.47(18)	O11-C23-C30-C29	-20.17(17)
C22-C23-C30-C19	27.1(2)	O11-C23-C30-C19	-149.57(14)
C22-C23-C30-C31	-88.6(2)	O11-C23-C30-C31	94.78(16)
C24-C29-C30-C23	16.66(17)	C28-C29-C30-C23	-174.83(17)
C24-C29-C30-C19	140.75(14)	C28-C29-C30-C19	-50.7(2)
C24-C29-C30-C31	-98.14(16)	C28-C29-C30-C31	70.4(2)
O8-C19-C30-C23	158.40(15)	C20-C19-C30-C23	-27.78(19)
O8-C19-C30-C29	40.8(2)	C20-C19-C30-C29	-145.37(14)
O8-C19-C30-C31	-82.89(18)	C20-C19-C30-C31	90.93(16)
C21-C20-C32-O9	-3.5(3)	C19-C20-C32-O9	179.51(17)
C21-C20-C32-C33	176.2(2)	C19-C20-C32-C33	-0.8(3)
C24-C25-C35-O12	177.61(19)	C26-C25-C35-O12	-2.0(3)
C24-C25-C35-C36	-2.5(4)	C26-C25-C35-C36	177.9(3)

Table S18. Anisotropic atomic displacement parameters (Å^2) for (*S*)-usnic acid (2).

The anisotropic atomic displacement factor exponent takes the form: $-2\pi^2[h^2 a^{*2} U_{11} + ... + 2 h k a^* b^* U_{12}]$

	U_{11}	U_{22}	U_{33}	U_{23}	U_{13}	U_{12}
O1	0.0519(7)	0.0376(6)	0.0462(6)	-0.0070(5)	-0.0053(5)	0.0067(5)
C1	0.0335(8)	0.0327(7)	0.0334(7)	0.0030(6)	0.0004(5)	-0.0030(6)
O2	0.0729(10)	0.0709(9)	0.0606(8)	-0.0020(7)	-0.0308(8)	0.0109(8)
C2	0.0379(8)	0.0375(8)	0.0334(7)	0.0016(6)	-0.0030(6)	0.0004(6)
O3	0.0744(10)	0.0617(8)	0.0475(7)	-0.0121(6)	-0.0236(7)	0.0045(7)
C3	0.0476(10)	0.0424(9)	0.0335(8)	-0.0026(6)	-0.0046(7)	-0.0004(7)
O4	0.0468(7)	0.0390(7)	0.0361(6)	-0.0035(4)	-0.0058(5)	0.0064(5)
C4	0.0547(11)	0.0418(9)	0.0377(8)	-0.0102(6)	-0.0076(7)	0.0048(8)
O5	0.0520(8)	0.0646(9)	0.0585(8)	0.0200(6)	-0.0097(6)	0.0066(7)
C5	0.0382(8)	0.0355(8)	0.0318(8)	-0.0026(5)	-0.0016(6)	0.0033(6)
O6	0.0507(8)	0.0780(11)	0.0343(6)	0.0124(6)	-0.0088(5)	-0.0061(7)
C6	0.0324(8)	0.0370(8)	0.0302(7)	0.0014(6)	-0.0007(5)	-0.0022(6)
O7	0.0655(9)	0.0406(7)	0.0468(7)	-0.0101(5)	-0.0069(6)	-0.0005(6)
C7	0.0324(8)	0.0457(9)	0.0356(7)	0.0084(6)	-0.0006(6)	-0.0034(6)
O8	0.0537(8)	0.0403(7)	0.0495(7)	-0.0110(5)	-0.0065(6)	0.0057(5)
C8	0.0333(8)	0.0545(10)	0.0305(7)	0.0085(6)	-0.0021(6)	-0.0097(7)
O9	0.0646(10)	0.0788(10)	0.0608(8)	0.0035(7)	-0.0173(7)	0.0158(8)
C9	0.0445(9)	0.0508(9)	0.0317(7)	-0.0016(6)	-0.0017(6)	-0.0113(7)
O10	0.0669(9)	0.0539(8)	0.0701(9)	0.0055(7)	-0.0074(8)	0.0198(7)

	U_{11}	U_{22}	U_{33}	U_{23}	U_{13}	U_{12}
C10	0.0391(8)	0.0392(8)	0.0336(7)	-0.0034(6)	0.0000(6)	-0.0069(6)
O11	0.0510(8)	0.0428(7)	0.0483(7)	-0.0117(5)	-0.0063(5)	0.0030(5)
C11	0.0343(8)	0.0360(8)	0.0301(7)	0.0002(5)	-0.0015(6)	-0.0034(6)
O12	0.0722(11)	0.1087(14)	0.0458(8)	-0.0154(8)	-0.0140(7)	-0.0031(10)
C12	0.0333(8)	0.0332(8)	0.0289(7)	0.0001(5)	-0.0013(5)	-0.0029(6)
O13	0.0516(8)	0.0813(11)	0.0425(7)	0.0156(7)	-0.0062(6)	-0.0006(7)
C13	0.0372(9)	0.0515(10)	0.0435(9)	0.0094(7)	0.0039(6)	-0.0065(7)
O14	0.0561(8)	0.0399(7)	0.0500(8)	-0.0057(5)	-0.0031(6)	0.0084(6)
C14	0.0407(9)	0.0458(9)	0.0441(8)	0.0077(7)	-0.0056(7)	-0.0008(7)
C15	0.0447(11)	0.0498(10)	0.0628(11)	0.0047(8)	-0.0077(8)	0.0073(8)
C16	0.0784(16)	0.0658(13)	0.0363(9)	-0.0081(8)	-0.0089(9)	-0.0147(11)
C17	0.0332(8)	0.0499(9)	0.0455(9)	0.0148(7)	0.0018(7)	-0.0006(7)
C18	0.0627(12)	0.0444(10)	0.0613(11)	0.0077(8)	-0.0038(10)	0.0097(9)
C19	0.0376(8)	0.0373(8)	0.0314(7)	-0.0005(5)	0.0047(6)	-0.0003(6)
C20	0.0404(9)	0.0402(9)	0.0350(7)	0.0015(6)	0.0020(6)	0.0022(7)
C21	0.0437(9)	0.0386(8)	0.0463(9)	0.0038(7)	0.0041(7)	0.0051(7)
C22	0.0491(10)	0.0334(8)	0.0541(10)	-0.0050(7)	0.0021(8)	0.0059(7)
C23	0.0403(9)	0.0335(8)	0.0424(8)	-0.0046(6)	0.0012(6)	-0.0012(6)
C24	0.0341(8)	0.0406(9)	0.0388(8)	-0.0034(6)	0.0017(6)	-0.0009(6)
C25	0.0371(8)	0.0594(10)	0.0359(8)	-0.0046(7)	0.0000(6)	-0.0038(7)
C26	0.0327(8)	0.0596(11)	0.0357(8)	0.0097(7)	0.0018(6)	-0.0042(7)
C27	0.0346(8)	0.0455(9)	0.0416(8)	0.0098(7)	0.0035(6)	-0.0012(7)
C28	0.0353(8)	0.0380(8)	0.0388(8)	0.0025(6)	0.0045(6)	-0.0006(6)
C29	0.0348(8)	0.0383(8)	0.0347(7)	0.0004(6)	0.0021(6)	-0.0006(6)
C30	0.0351(8)	0.0332(8)	0.0351(8)	-0.0002(6)	0.0031(6)	-0.0004(6)
C31	0.0387(9)	0.0517(10)	0.0492(10)	0.0107(7)	0.0085(7)	-0.0022(8)
C32	0.0470(10)	0.0592(11)	0.0370(8)	0.0038(7)	-0.0023(7)	0.0023(9)
C33	0.0777(16)	0.0686(13)	0.0500(11)	-0.0100(9)	-0.0220(11)	-0.0005(12)
C34	0.0490(11)	0.0498(11)	0.0613(11)	0.0169(9)	0.0002(8)	0.0036(8)
C35	0.0452(10)	0.0860(15)	0.0442(9)	-0.0177(9)	-0.0053(8)	-0.0029(10)
C36	0.120(3)	0.116(3)	0.092(2)	-0.0662(19)	-0.047(2)	0.046(2)

Table S19. Hydrogen atomic coordinates and isotropic atomic displacement parameters (Å^2) for (*S*)-usnic acid (2).

	x/a	y/b	z/c	U(eq)
H3	0.132(4)	0.6958(15)	0.0144(14)	0.080000
H4	0.4366	0.5758	0.0833	0.054000
H6	0.837(4)	0.5856(15)	0.4088(13)	0.067(8)
H7	0.432(4)	0.8116(15)	0.2643(15)	0.074(8)
H10	0.738(4)	0.4470(16)	0.5410(15)	0.080000
H13	0.028(4)	0.6014(17)	0.1653(16)	0.088(10)
H13A	0.7519	0.7255	0.1520	0.066000
H13B	0.6688	0.7979	0.1676	0.066000
H13C	0.6306	0.7598	0.1011	0.066000
H14	0.274(3)	0.6845(12)	0.4391(12)	0.053(7)
H15A	0.0196	0.8270	0.1530	0.079000
H15B	-0.0428	0.8519	0.0839	0.079000
H15C	0.1346	0.8731	0.1083	0.079000
H16A	0.6089	0.8137	0.4118	0.090000
H16B	0.7679	0.7726	0.4338	0.090000
H16C	0.5927	0.7484	0.4582	0.090000

	x/a	y/b	z/c	U(eq)
H18A	0.7836	0.5031	0.2186	0.084000
H18B	0.6269	0.4728	0.2542	0.084000
H18C	0.8028	0.4418	0.2694	0.084000
H22	0.5202	0.3945	0.3890	0.055000
H31A	0.0856	0.4991	0.4408	0.070000
H31B	0.1221	0.5634	0.4867	0.070000
H31C	0.2058	0.4907	0.5008	0.070000
H33A	0.7151	0.6481	0.5482	0.098000
H33B	0.7572	0.6139	0.6163	0.098000
H33C	0.5719	0.6266	0.5963	0.098000
H34A	-0.0012	0.7607	0.3402	0.080000
H34B	-0.0546	0.7459	0.2674	0.080000
H34C	0.1198	0.7786	0.2822	0.080000
H36A	0.1828	0.4127	0.2302	0.164000
H36B	0.3461	0.4439	0.2004	0.164000
H36C	0.2049	0.4172	0.1537	0.164000

Table S20. Hydrogen bond distances (Å) and angles (°) for (*S*)-usnic acid (2).

	Donor-H	Acceptor-H	Donor-Acceptor	Angle
O3-H3···O2	1.19(3)	1.26(3)	2.406(2)	159.(3)
O6-H6···O5	1.03(3)	1.60(3)	2.534(2)	149.(2)
O7-H7···O1	0.97(3)	1.70(3)	2.6637(18)	178.(3)
O9-H10···O10	1.21(3)	1.25(3)	2.415(2)	158.(3)
O13-H13···O12	1.09(3)	1.47(3)	2.520(3)	159.(3)
O14-H14···O8	0.88(3)	1.83(3)	2.6871(19)	165.(2)
